# Efficacy of Mesenchymal Stromal Cell Therapy for Acute Lung Injury in Preclinical Animal Models: A Systematic Review

**DOI:** 10.1371/journal.pone.0147170

**Published:** 2016-01-28

**Authors:** Lauralyn A. McIntyre, David Moher, Dean A. Fergusson, Katrina J. Sullivan, Shirley H. J. Mei, Manoj Lalu, John Marshall, Malcolm Mcleod, Gilly Griffin, Jeremy Grimshaw, Alexis Turgeon, Marc T. Avey, Michael A. Rudnicki, Mazen Jazi, Jason Fishman, Duncan J. Stewart, Canadian Critical Care Translational Biology Group

**Affiliations:** 1 Department of Medicine (Division of Critical Care), University of Ottawa, Ottawa, Ontario, Canada; 2 The Ottawa Hospital Research Institute, Ottawa, Ontario, Canada; 3 Department of Epidemiology and Community Medicine, University of Ottawa, Ottawa, Ontario, Canada; 4 Department of Anesthesiology, University of Ottawa, Ottawa, Ontario, Canada; 5 Department of Surgery and Critical Care Medicine, Keenan Research Centre of the Li Ka Shing Knowledge Institute, St. Michaels Hospital, The University of Toronto, Toronto, Ontario, Canada; 6 Centre for Clinical Brain Sciences, The University of Edinburgh, Edinburgh, Scotland, United Kingdom; 7 Department of Medicine, University of Ottawa, Ottawa, Ontario, Canada; 8 Department of Anesthesiology and Critical Care Medicine, Division of Critical Care Medicine, Université Laval, Laval, Québec City, Québec, Canada; 9 Population Health and Optimal Health Practice Research Unit (Trauma—Emergency—Critical Care Medicine), CHU de Québec Research Center, CHU de Québec (Hôpital de l'Enfant-Jésus), Laval, Québec City, Québec, Canada; 10 Department of Cell and Molecular Medicine, University of Ottawa, Ottawa, Ontario, Canada; 11 University of Ottawa, Ottawa, Canada; University of Illinois College of Medicine, UNITED STATES

## Abstract

The Acute Respiratory Distress Syndrome (ARDS) is a devastating clinical condition that is associated with a 30–40% risk of death, and significant long term morbidity for those who survive. Mesenchymal stromal cells (MSC) have emerged as a potential novel treatment as in pre-clinical models they have been shown to modulate inflammation (a major pathophysiological hallmark of ARDS) while enhancing bacterial clearance and reducing organ injury and death. A systematic search of MEDLINE, EMBASE, BIOSIS and Web of Science was performed to identify pre-clinical studies that examined the efficacy MSCs as compared to diseased controls for the treatment of Acute Lung Injury (ALI) (the pre-clinical correlate of human ARDS) on mortality, a clinically relevant outcome. We assessed study quality and pooled results using random effect meta-analysis. A total of 54 publications met our inclusion criteria of which 17 (21 experiments) reported mortality and were included in the meta-analysis. Treatment with MSCs, as compared to controls, significantly decreased the overall odds of death in animals with ALI (Odds Ratio 0.24, 95% Confidence Interval 0.18–0.34, I^2^ 8%). Efficacy was maintained across different types of animal models and means of ALI induction; MSC origin, source, route of administration and preparation; and the clinical relevance of the model (timing of MSC administration, administration of fluids and or antibiotics). Reporting of standard MSC characterization for experiments that used human MSCs and risks of bias was generally poor, and although not statistically significant, a funnel plot analysis for overall mortality suggested the presence of publication bias. The results from our meta-analysis support that MSCs substantially reduce the odds of death in animal models of ALI but important reporting elements were sub optimal and limit the strength of our conclusions.

## Introduction

The Acute Respiratory Distress Syndrome (ARDS) was first recognized in the 1960s as a clinical syndrome of severe acute respiratory failure. Although definitions have been recently revised, the consistent hallmarks are the acuity of presentation, and the presence of severe hypoxemia and bilateral pulmonary infiltrates[1]. It is a devastating clinical condition with approximately 200 000 new cases identified per year in the United States and a case fatality rate of approximately 30–40%[1]. Those who do recover experience a significant decrease in quality of life with long term physical, physiological, and emotional dysfunction[2]. Over the last several decades many novel therapeutics have been evaluated for the treatment of ARDS yet none have proven efficacious, and thus supportive care strategies including institution of antibiotics, low tidal volume mechanical ventilation, and fluid restriction remain the mainstays of therapy[1,3]. Critiques of novel therapeutics have highlighted inadequate clinical trial design and conduct, and more recently inadequacies of preclinical design and conduct as reasons for failure of translation[4–6]. Recent advances in the study and knowledge of stem cells has allowed for stem cell therapy to emerge as a potential novel therapeutic for the treatment of ARDS. Mesenchymal stromal cells (MSCs) are immune-modulatory and pre-clinical studies in animal models of acute lung injury (ALI) (the pre-clinical correlate of human ARDS) suggest MSCs reduce inflammation, augment tissue repair, enhance pathogen clearance, and reduce death[7–11]. This systematic review was conducted to better inform a decision to translate MSC therapy for pre-clinical ALI into a human clinical trial. We aimed to systematically summarize all pre-clinical studies to examine the efficacy of this treatment as compared to a diseased control group across different animal and ALI induction models; MSC origin, source and preparation; and the clinical relevance of ALI models on the clinically relevant outcome death.

## Results

### Study Characteristics

**Fig 1:**
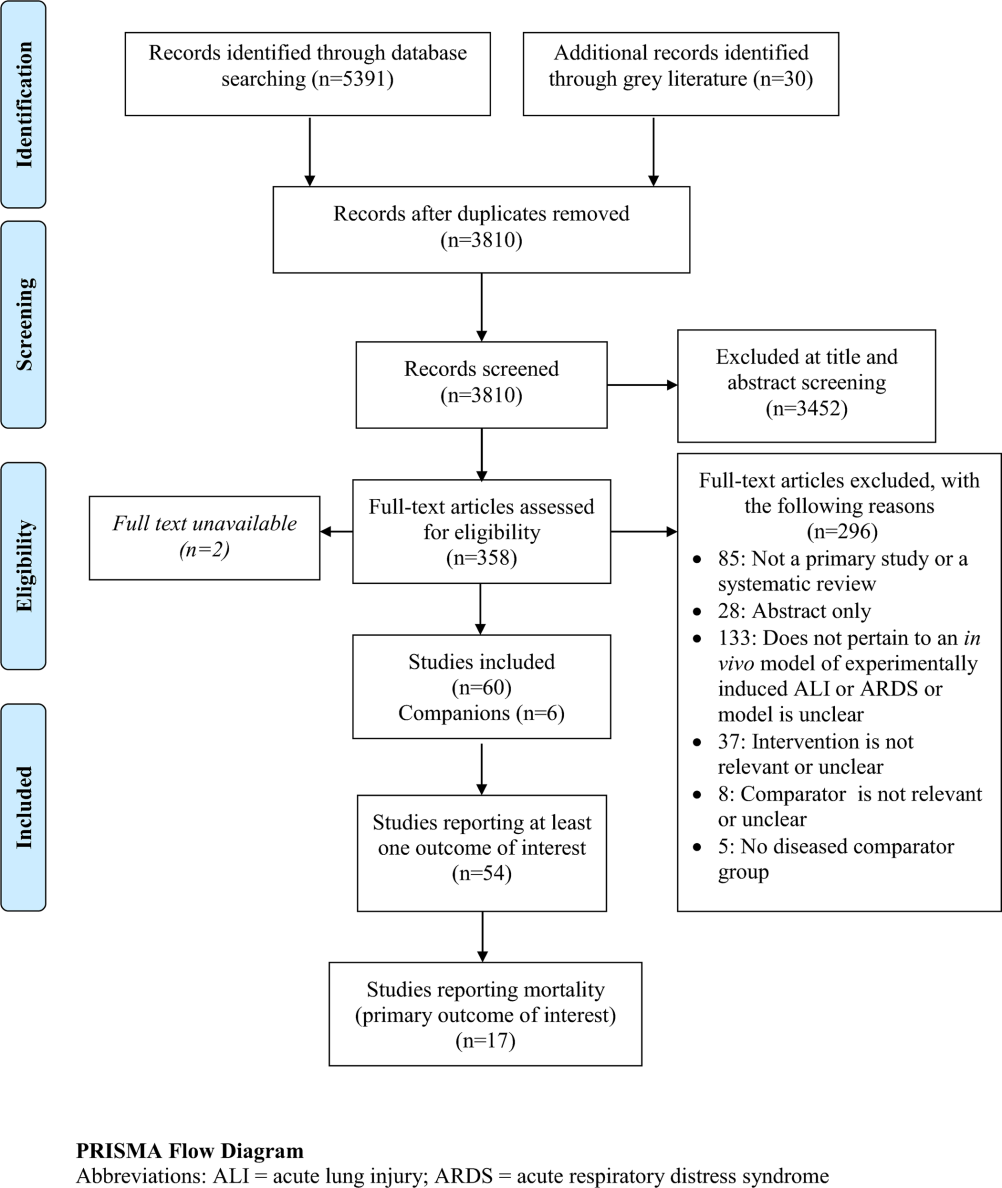
PRISMA flow diagram.

Our search yielded 3810 citations to screen. After preliminary screening a total of 358 citations were pulled for full text review; 54 publications met our pre-defined eligibility criteria and were included in the review (**Fig 1**)[7,10–62] These reported 70 experiments (**Table 1, S1 File, S1 Table**), of which 21(from 17 publications) reported our primary outcome death and were included in the meta-analysis.[7,10,15–18,29,31,32,34,37,41,43,44,46,47,59]

**Table 1:** Summary of baseline characteristics.

Group	Subgroup Analysis	All Experiments (n = 70) N (%)	Experiments that Reported |Mortality (n = 21) N (%)
Animal Model	Mouse	32 (46)	13 (62)
	Rat	33 (47)	8 (38)
	Rabbit	5 (7)	0 (0)
Gender	Male	39 (56)	13 (62)
	Female	16 (23)	5 (24)
	Not reported	11 (16)	3 (14)
	Mixed	4 (6)	0 (0)
ALI Experimental Model	Direct Infection/Inflammation	20 (29)	5 (24)
	Indirect Infection/Inflammation	22 (31)	11 (52)
	Direct Chemical Injury	7 (10)	2 (10)
	Indirect Chemical Injury	7 (10)	2 (10)
	Combination	1 (1)	1 (5)
	Trauma	6 (9)	0 (0)
	Pulmonary ischemia/reperfusion	2 (3)	0 (0)
	Ventilation	5 (7)	0 (0)
MSC Origin*	Syngeneic	38 (54)	9 (43)
	Xenogenic	26 (37)	9 (43)
	Allogeneic	6 (9)	3 (14)
	Autologous	2 (3)	1 (5)
MSC Source	Bone Marrow	54 (77)	13 (62)
	Adipose Tissue	8 (11)	3 (14)
	Umbilical Cord	8 (11)	5 (24)
MSC Preparation	Fresh#	17 (24)	4 (19)
	Cryopreserved	3 (4)	2 (10)
	Unclear	50 (71)	15 (71)
Route of Administration*	Intratracheal	10 (14)	6 (29)
	Intravenous	52 (74)	12 (57)
	Intraperitoneal	4 (6)	3 (14)
	OA	4 (6)	0 (0)
	IPL	1 (1)	0 (0)
	IM	2 (3)	0 (0)
Timing of Administration*	0 h	14 (20)	2 (10)
	>0 h to ≤1 h	23 (33)	8 (38)
	>1 – ≤6 h	23 (33)	11 (52)
	>6 h	12 (17)	3 (14)
	Multiple Times	10 (14)	0 (0)
	Unclear	2 (3)	0 (0)
	Not Reported	1 (1)	0 (0)
Resuscitation	None	62 (89)	15 (71)
	Antibiotics	1 (1)	1 (5)
	Fluid	5 (7)	3 (14)
	Fluid and antibiotics	2 (3)	2 (10)
Control Group*	Fibroblast	15 (21)	5 (24)
	Cell	1 (1)	0 (0)
	Normal saline	22 (31)	8 (38)
	Phosphate buffered saline	28 (40)	9 (43)
	Vehicle	7 (10)	1 (5)
	Medium	4 (6)	2 (10)
	Fibroblast Conditioned Media	1 (1)	0 (0)
	Unclear	1 (1)	1 (5)
	Nothing	9 (13)	1 (5)

*Percentages don’t equal 100% as some experiments are multi-arm

#Fresh includes thawed and cultured MSCs, in addition to newly extracted MSCs

Of the 70 experiments the majority originated from Asia (51%, n = 36)[10,12,15–18,20–22,26–28,32–35,37–39,43,48,49,52–56,58–60,62], with 27% (n = 19) from North America[7,11,13,24,25,29,36,40–42,47,50,51,57], 11% (n = 8) from Europe[19,23,30,44,46], 4% (n = 3) from Australia or New Zealand[14,45], and 6% (n = 4) from a collaboration between countries (n = 2 Italy/United States, n = 2 Canada/Brazil) (**S1 Table**)[31,61]. Rats and mice were studied in 47% (n = 33)[12,15–21,23,24,26–28,30,32,34–36,40,42,43,48,52,53,56,60] and 46% (n = 32)[7,10,11,13,14,22,25,29,31,33,37,41,44–47,49–51,57–59,61] of experiments, respectively, while 7% (n = 5) of experiments were conducted on rabbits[38,39,54,55,62]. Two experiments included animals with compromised immune systems (severe combined immune-deficient (SCID) mice)[14,45]. Several methods were used to induce ALI in the animals. These included direct (29%, n = 20)[7,10,11,13,21,22,25,29,33,37,49,50,55,57,58,61] and indirect (31%, n = 22) [12,15,17,31,32,34,40–42,46,47,51–53,59,61] lung infection or inflammation, direct (10%, n = 7)[14,43–45,48,56] and indirect (10%, n = 7) [18,20,26,28,60] chemical induction, trauma (9%, n = 6) [24,36,38,39,54,62], induction by the ventilator (7%, n = 5)[19,23,30], pulmonary ischemia and reperfusion (3%, n = 2)[27,35], and a combination of the above methods (1%, n = 1)[16]. Of the 11 experiments that were infectious pre-clinical ALI models[7,10,11,17,31,41,46,47,59], 17% (n = 2) involved the administration of fluids to the animals[41,59], 8% (n = 1) the administration of antibiotics[10], and 17% (n = 2) the administration of both fluids and antibiotics[41,47].

To treat pre-clinical ALI, the majority of experiments (54%; n = 38) used syngeneic MSCs[7,16,19–30,32–34,38,39,41,43,48–51,54,56,57,61,62], 37% (n = 26) used xenogenic cells[10–15,18,31,37,40,42,45,46,52,53,57–59], 9% (n = 6) allogeneic cells [36,44,46,47,55,60], and 3% (n = 2) used autologous MSCs[17,35]. The source of MSCs included bone marrow (77%, n = 54)[7,11,13,14,16,19–34,36,38–44,47–51,53–56,59–62], adipose tissue (11%, n = 8)[12,17,35,46,52,57,58], and umbilical cord (11%, n = 8)[10,15,18,37,45]. MSCs were most often administered as a single dose (89%, n = 62)[7,10–16,18–29,31–34,36–56,58–62] and via an intravenous route (74%, n = 52)[12–16,18,20,22–24,26–28,30–36,38,39,41,43,45,47–56,58–62]. In 71% (n = 50) of the experiments it was unclear if the MSCs infused were fresh or cryopreserved[7,10,12,15,16,19–30,32–34,36–39,43–49,51–56,58–62]; 24% (n = 17) indicated the cells infused were fresh[11,13,14,17,31,35,40–42,50,57] and 4% (n = 3) indicated the cells were cryopreserved[18]. The dose of MSCs administered varied between 5.0 x 10^4^ and 3.6 x 10^7^. The majority (84%, n = 59) of experiments included 1 MSC intervention arm[7,10–19,21–45,48–52,54–56,58–62]. Eleven (16%) of the experiments included more than one MSC arm[13,18,20,32,46,47,53,57]. Phosphate buffered saline (40%, n = 28)[7,10,11,13,21–23,26,29–31,33,37–39,42,44,47,54,58,59,62], normal saline (31%, n = 22)[12,14–18,34,36,41,45,49,50,52,55,61], and fibroblasts (21%, n = 15)[10,13,15,23–25,29–31,45,47,51] were the most common control agents used in the experiments.

### Risk of Bias

Risk of bias[63] (**S2 Table**) was evaluated for the 21 experiments that reported death and were included in the meta-analysis [7,10,15–18,29,31,32,34,37,41,43,44,46,47,59]. None of the 21 experiments were considered low risk of bias across all domains and none were considered low risk of bias for each of randomization, allocation concealment, and blinding. Although 48% (n = 10)[10,15,17,18,32,34,41,59] of the experiments indicated the group allocation was randomized, none described the randomization procedures or that personnel conducting the experiments were blinded to the study groups [7,10,15–18,29,31,32,34,37,41,43,44,46,47,59]. For 71% (n = 15) of the experiments, animals were either allowed to die or assessors for the mortality outcome were blinded to the study groups (low risk)[7,10,16,18,29,31,34,41,44,46,47]; blinding of the mortality outcome was unclear for the remainder (29%, n = 6)[15,17,32,37,43,59]. For assessment of the 'incomplete outcome data risk of bias domain, most of the experiments were either of unclear or high risk of bias (67%, n = 14)[7,10,29,31,37,41,43,44,46,47,59], while 33% (n = 7) were low risk[15–18,32,34]. The death outcome was considered selectively reported (high risk of bias) in 10% (n = 2)[43,44] of publications, with 90% of studies being assessed as low risk of bias (n = 19)[7,10,15–18,29,31,32,34,37,41,46,47,59]. Other potential sources of bias (source of funding, conflict of interest and pre-specified sample size calculations) were evaluated; 1 publication was considered at low risk of bias for all three variables[17] (**S3 Table**).

### Meta-Analysis: Primary Outcome Mortality

Mortality was reported as an outcome in 33% (n = 23) of the 70 experiments[7,10,15–19,29,31,32,34,37,41,43,44,46,47,59,60]. One experiment was not included as it did not have an ALI diseased control group as a comparison[19], and another experiment did not present data in a form that allowed for number of events to be analyzed[18]. Therefore, data from 21 experiments (17 publications) were included in the meta-analysis [7,10,15–18,29,31,32,34,37,41,43,44,46,47,59].

**Fig 2:**
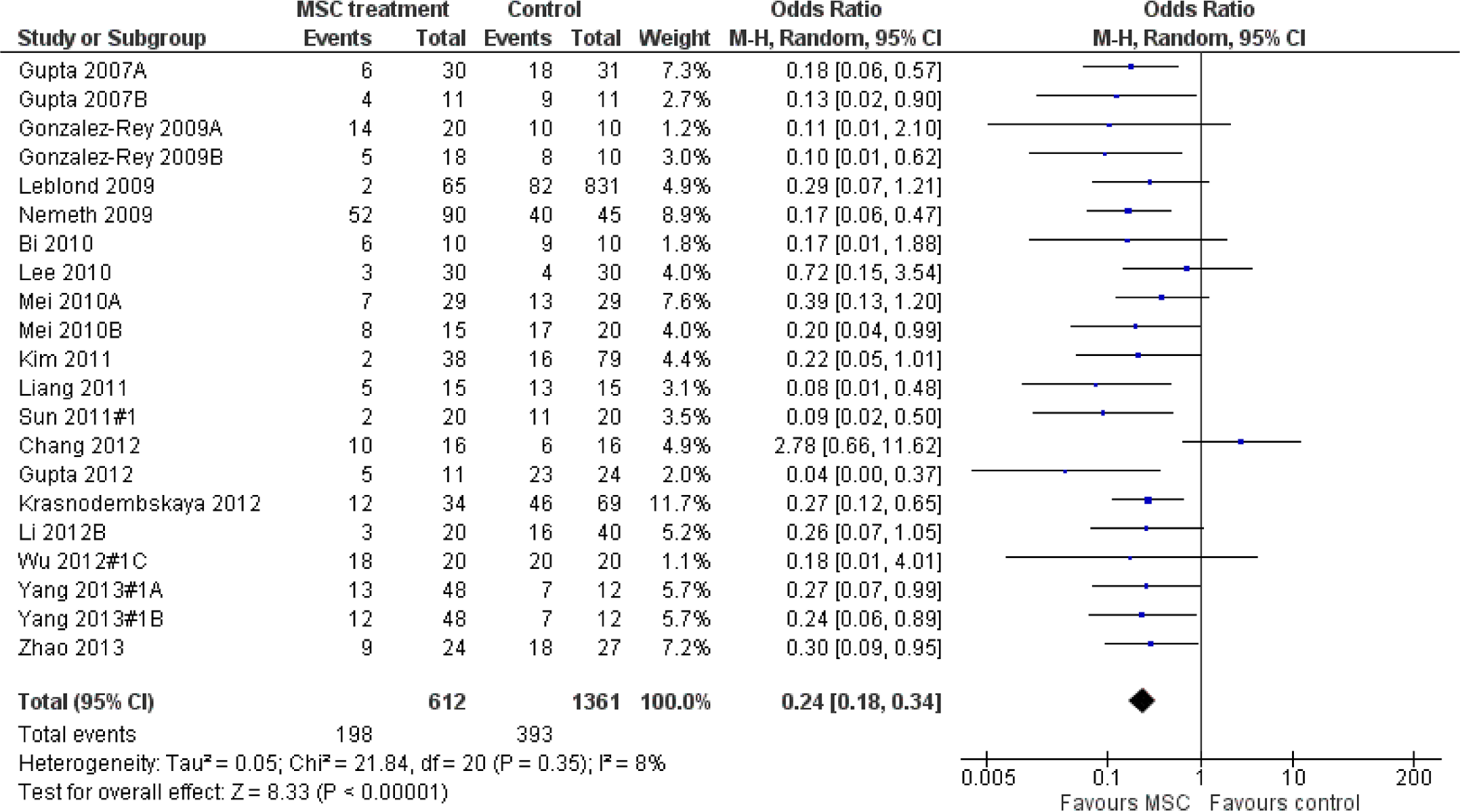
Forest plot of mesenchymal stem cellson the odds of mortality in preclinical models of acute lung injury. Letters indicate two separate mortality experiments within one publication.

**Fig 3:**
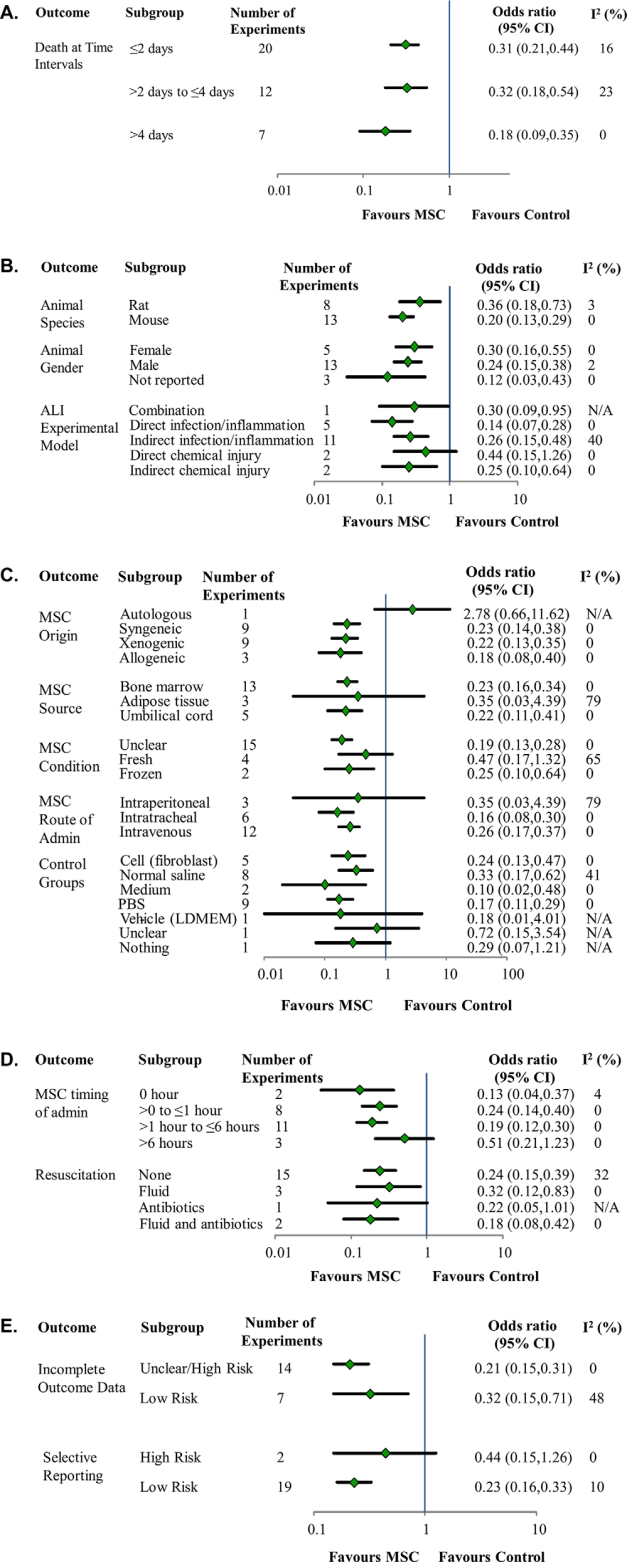
Subgroup analyses of mesenchymal stem cellson the odds of mortality in preclinical models of acute lung injury. **Fig 3A**: Forest plot of mesenchymal stem cells on the odds of mortality at a priori determined time points. **Fig 3B**: Forest plot of mesenchymal stem cells on the odds of mortality according to animal species, gender and experimental model of acute lung injury. **Fig 3C**: Forest plot of mesenchymal stem cells on the odds of mortality according to MSC origin, source, preparation, and route of administration, as well as comparator control groups. Subgroup analyses conducted to examine the robustness of the treatment effect according to the clinical relevance of the ALI model (timing of MSC administration in relation to ALI induction and resuscitation of the animals) (**Fig 3D**) indicated a reduction in the odds of death regardless of the timing of administration of the cells, although the protective effect of MSCs appeared less the longer the delay in treatment initiation. There were no significant differences in the treatment effect of MSCs with more clinically relevant animal models (e.g. use of antibiotics, resuscitation fluid, or the combination of resuscitation fluid and antibiotics). Analyses conducted according to selective outcome reporting and incomplete outcome reporting did not reveal substantial differences in the estimate of effect (**Fig 3E**). **Fig 3D**: Forest plot of mesenchymal stem cells on the odds of mortality according to timing of MSC administration and method of resuscitation. **Fig 3E**: Forest plot of mesenchymal stem cells on the odds of mortality according to domains of the Cochrane Risk of Bias.

Treating pre-clinical ALI with MSCs significantly decreased the overall odds of death (Odds Ratio (OR) 0.24, 95% Confidence Interval (CI) 0.18–0.34) compared to diseased controls without substantial heterogeneity (I^2^ 8%) (**Fig 2**). The odds of death were also reduced when examined at pre-specified death time points (death at less than or equal to 2 days (OR 0.31, 95% CI 0.21–0.44, I^2^ 16%), between 2 to 4 days (OR 0.32, 95% CI 0.18–0.54, I^2^ 23%), and greater than 4 days (OR 0.18, 95% CI 0.09–0.35, I^2^ 0%) (**Fig 3A**). The treatment effect was examined for pre-specified sub groups (animal gender and species, ALI experimental model, MSC origin and source, route of administration, and MSC preparation) (**Fig 3B and 3C**). All of these sub groups suggested a similar protective treatment effect of MSCs with exception of one “MSC origin” subgroup that originated from one experiment[17]. In this experiment autologous adipose-derived MSCs were administered to a rat model of indirect infection model via an intraperitoneal route (OR for death 2.78, 95% CI 0.66–11.62).

### Publication Bias

**Fig 4:**
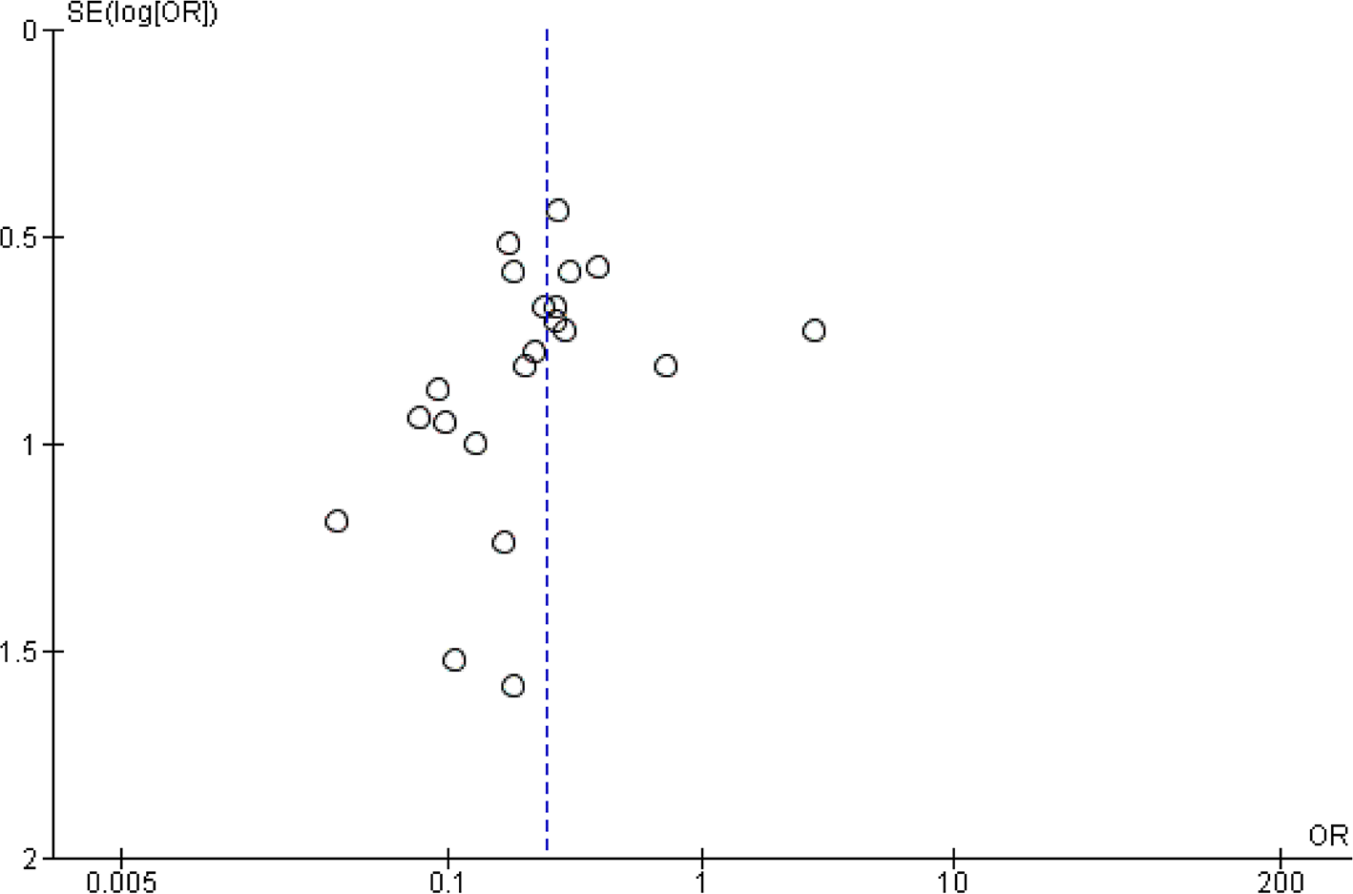
Funnel plot of standard error by log odds ratio for overall mortality indicates the possibility of publication bias.

Visually, the funnel plot suggested some degree of asymmetry (e.g., possible publication bias) although this was not confirmed by Egger regression (p-value of 0.16) (**Fig 4**).

### MSC Characterization

We evaluated reporting of standard MSC characterization criteria according to the International Society for Cellular Therapy guidelines[64] for the 18 of 54 publications that included the administration of human (xenogenic) MSCs (**S4 Table**)[10–15,18,31,32,37,40,42,45,46,52,57–59]. None of these reported all three criteria (plastic adherence, differentiation potential, and cell surface antigen expression). The ability for MSCs to adhere to plastic was reported in 39% (n = 7)[10,12,15,37,45,57,59] of publications. MSC differentiation into 3 cell lineages (adipocytes, osteoblasts and chondroblasts) was reported in 6% (n = 1)[10]; whereas differentiation into one or two cell lines (adipocytes and or osteoblasts) was reported in 6% (n = 1)[45] and 28% (n = 5[15,37,42,46,57] of the publications respectively. One publication (6%) reported the proportion of cell surface antigen expression in accordance with the recommended International Society for Cellular Therapy guidelines[18]. MSCs were purchased from another manufacturer in 39% (n = 7) publications[11,13,14,18,31,40,42]; none of these reported all three MSC characterization criteria according to Dominici et al[64].

## Discussion

To the best of our knowledge this is the first systematic review to examine the effect of MSCs on mortality in pre-clinical ALI. The results show that treatment of pre-clinical ALI with MSCs reduces the odds of death compared to untreated diseased control animals. This was true for overall mortality, as well as mortality at pre-defined time intervals (≤2, > 2 to ≤ 4, and > 4 days). Subgroup analyses according to the species, gender, and ALI experimental model; MSC origin, source, route and preparation of MSCs; and clinical relevance of the ALI model (timing of MSC administration, administration of fluids and/or antibiotics in relevant models) all found MSCs, as compared to controls, were associated with reductions in death. Visualization of funnel plots suggested the presence of publication bias, although this was not statistically significant.

Results of our systematic review suggest that MSCs are beneficial across a range of animal models and experimental conditions and it is encouraging that the protective effects of MSCs appeared to be sustained even when more clinically relevant animal models were studied. For example, MSCs reduced death as compared to diseased controls even when the initiation of MSC therapy was delayed to longer than 6 hours post pre-clinical ALI induction. Although not statistically significant, the magnitude of reduction in the odds of death was less the longer MSC administration was delayed suggesting at least in animal models of ALI that time to treatment may impact the magnitude of effect. Antibiotics were administered in only 3 of the 11 experiments that were infectious models of ALI. The conduct of clinically relevant experiments is important in the evaluation of MSC efficacy especially when the therapeutic approach is being considered for clinical evaluation.

We found that some characteristics of study reporting were inadequate. For example, reporting on the 3 standardized characterization criteria for human MSCs[64] was poor; no publication reported on all 3 criteria. Investigators should provide more detailed reporting on the characterization of the MSCs to enable adequate comparisons across different research experiments as well as the conduct of future meta-analyses according to these variables. Empirical evidence suggests that use of reporting guidelines are effective to improve the completeness of reporting[65] and the ARRIVE (Animal Research: Reporting of *In Vivo* Experiments) guidelines are one way to help improve the reporting of animal studies[66].

Reporting of risk of bias domains[63] was also generally poor. None of the 21 experiments were considered a low risk of bias for all domains. Reporting these domains in pre-clinical studies is important as the methodological shortcoming that bias the treatment effects in clinical trials[67,68] may also apply to pre-clinical studies. Some pre-clinical interventional research in stroke and emergency medicine suggest that methodological weaknesses may be associated with inflations in the estimates of the effect size for different treatments[69]. As one example, although approximately 50% of experiments included in our review were reported as randomized, none explained the randomization method and none indicated that the allocation lists were concealed from personnel involved in the conduct of the experiment. Both of these domains are important measures of internal validity in randomized trials. We submit that when comparative efficacy pre-clinical MSC studies are being conducted, they should aim for the same methodological rigor as clinical trials to ensure a non-biased estimate of the true treatment effect.

Our systematic review has several strengths. We included a systematic and transparent search of the literature and involved an independent review of our search strategy to ensure identification of all eligible citations. We reported a primary outcome that is relevant in the clinical domain and several pre-specified sub group analyses to examine heterogeneity of the treatment effect. However, our review is limited by the publication of pre-clinical studies that are available in the public domain. Furthermore, since visualization of the funnel plot and the Egger's regression test suggested some evidence of publication bias, we cannot rule out that the treatment effect of MSCs could be less strong, not effective, or harmful in certain animal sub groups of unpublished data. However, the consistency of effect of MSCs observed across several subgroup analyses in the published literature is encouraging and we included a systematic search that was PRESS reviewed[70] to identify both published and unpublished studies.

In conclusion, MSCs appear substantially to reduce death in pre-clinical models of ALI and across many sub groups. Our review suggests that this therapy could provide a potential future treatment for many different types of acute lung injury and provides supportive evidence for moving toward their evaluation in human clinical trials. However, we also found that certain reporting elements related to risk of bias domains and MSC characterization were inadequate which could be improved substantially with use of a pre-clinical reporting guideline such as ARRIVE (Animal Research: Reporting of *In Vivo* Experiments).

## Methods

Our protocol was registered on the CAMARADES website in March 2014 (http://www.dcn.ed.ac.uk/camarades/files/MSCs%20in%20preclinical%20models%20of%20acute%20lung%20injury.pdf) and published in Systematic Reviews[67]. Our methods are in accordance to the Preferred Reporting Items for Systematic Reviews and Meta-Analyses (PRISMA)[71] and are briefly described here (**S5 Table**).

### Inclusion/Exclusion Criteria

We included randomized and non-randomized studies that examined an in vivo model of experimentally induced ALI compared to a diseased control group, where MSCs were administered during or after the experimental induction of ALI. We excluded neonatal animal ALI models (i.e., mice or rats less than 10 days of age); MSC prevention studies (i.e. mesenchymal stromal cells administered prior to lung injury); studies where MSCs were differentiated, altered, or engineered to over or under express particular genes; or where MSCs were administered with another therapy or cell type (not including co-interventions such as antibiotics or steroids).

### Literature Search

We searched Ovid MEDLINE (1946 onwards), Ovid MEDLINE In-Process & Other Non-Indexed Citations (1946 onwards), and Embase Classic + Embase (using the OVID platform) (1947 onwards), as well as BIOSIS (1926 onwards) and Web of Science (using Web of Knowledge) (1900 onwards) until June 5, 2013 with no language restrictions. The search was designed by an information specialist and used key words such as “Mesenchymal Stem Cells”, “Adult Respiratory Distress Syndrome”, “Acute Lung Injury”, and “Animal Experimentation” and was modified according to the database searched for best results. Prior to execution, the MEDLINE search strategy was peer reviewed using the PRESS tool (Peer Review of Electronic Search Strategies)[70]. All references were de-duplicated manually within Reference Manager prior to screening.[72]

A grey literature search of targeted conferences and animal research organizations not covered in these electronic databases was also performed. Additional references were sought through hand-searching the bibliographies of reviews and a random sample of included studies.

### Screening

All citations were screened for inclusion with 2 trained systematic reviewers using a liberal accelerated method[73]. Screening occurred at two levels; title and abstracts were screened at level 1 and full-texts were screened at level 2. Disagreements were resolved by consensus and by consultation with a third member of the team when necessary.

### Data Extraction

Data was collected on general study characteristics(e.g. study design, region of origin, funding sources, etc.), MSC characterization criteria (e.g. MSC source, tissue origin, dose, etc.), risk of bias (e.g. random sequence generation, blinding, etc.), and outcome measurements including death (primary) and serum/plasma inflammatory cytokines, organ function, and bacterial clearance for infectious ALI models (secondary). All data extracted were verified by a second reviewer. Disagreements during extraction were resolved by consensus or third party consultation. Data for the mortality outcome was collected as number of events. For two experiments, the sample size of the animals studied was presented as a range (n = 11–12[29]and n = 8–10[46]); authors were contacted for clarification and both responded with the actual sample size. When the number of deaths could not be ascertained, the report was excluded from further analysis (n = 1)[60].

### Primary and Secondary Endpoints

Overall mortality was defined as death that was reported at the latest follow up point. Mortality was also reported at pre-specified time intervals: less than 2 days, between 2–4 days, and greater than 4 days after induction of ALI to quantify the effect of MSC treatment in pre-clinical ALI over time. This paper presents the results of the primary outcome analyses. The secondary outcome analyses will be reported in a future paper.

### Assessing Risk of Bias

Risk of bias was assessed using the Cochrane Risk of Bias tool for experiments that reported mortality[63]. The Cochrane assessment examines seven domains of bias: 1) Sequence generation, 2) Allocation concealment, 3) Blinding of participants and personnel, 4) Blinding of outcome assessors, 5) Incomplete outcome data, 6) Selective outcome reporting, and 7) Other sources of bias. Other sources of bias were assessed based on the source of funding, a conflict of interest statement and sample size determination. Assessment was done in duplicate, with disagreements resolved through consensus or consultation with a third party.

Since death maybe a subjective assessment in pre-clinical experiments when it is defined according to physiological endpoints[74], for assessment of the 'blinding of the outcome' risk of bias domain we considered the experiment to be of low risk if animals were allowed to die or if it was reported in the manuscript that the assessors of this outcome were blinded to the study groups (applicable when death was defined according to a physiological endpoint).

### Assessing MSC Characterization Criteria

Experiments that included the administration of human MSCs were assessed for the recommended minimal characterization criteria for multi-potent human MSCs, as defined by the International Society for Cellular Therapy (ISCT)[64]. The ISCT proposed a total of three criteria to define human MSCs: 1) the ability for MSCs to adhere to plastic in standard tissue culture flasks, 2) demonstration of multipotent differentiation potential into osteoblasts, adipocytes and chondroblasts under standard in vitro differentiating conditions, and 3) expression of specific surface antigens on the MSCs: ≥ 95% of the cells must express CD105, CD73 and CD90; ≤ 2% can express CD45, CD34, CD14 or CD11b, CD79α or CD19, and HLA-DR[64].

### Analysis

Mortality data was pooled across included studies using a random effects model and described according to odds ratios and 95% confidence intervals. To allow for meta-analysis, when an experiment included multiple MSC intervention arms or diseased control arms, the mortality data were pooled into a single intervention and control value. Forest plots were utilized to visualize the data. Statistical heterogeneity of the included studies was assessed using the I^2^ test with 95% confidence intervals. Publication bias was assessed visually using a funnel plot and analytically using Egger’s regression test.

Planned subgroup analyses were performed to examine the heterogeneity of the treatment effect of MSCs on overall mortality (**Table 1**). Pre-specified subgroups included analyses according to the animal model and gender; ALI model; MSC origin, source, and preparation, route of administration; clinical relevance of the model (timing of MSC administration in relation to ALI induction and resuscitation (as defined by administration of fluids, antibiotics or fluids and antibiotics for infectious ALI models)), and risk of bias domains.

## Supporting Information

S1 File References of included studies. (DOCX)

S1 Table General characteristics of all included experiments. (DOCX)

S2 Table Cochrane risk of bias assessment. (DOCX)

S3 Table Cochrane “other” risk of bias assessment. (DOCX)

S4 Table Criteria for identification of cell population as human mesenchymal stromal cells. (DOCX)

S5 Table PRISMA checklist. (DOCX)

## Abbreviations

ALI: acute lung injury
ARDS: Acute respiratory distress syndrome
CI: Confidence interval
MSC: Mesenchymal stromal cells
OR: Odds ratio
SCID: Severe combined immunodeficient
