# Supplementary material for: Efficacy of Mesenchymal Stromal Cell Therapy for Acute Lung Injury in Preclinical Animal Models: A Systematic Review
Source: PLoS One. 2016 Jan 28;11(1):e0147170. doi: 10.1371/journal.pone.0147170 (PMC4731557; doi:10.1371/journal.pone.0147170)
Supplement: S1 File — (DOCX) [file pone.0147170.s001.docx]

**S1 File**: References for 54 studies that meet inclusion criteria

1. Gupta N, Su X, Popov B, Lee JW, Serikov V, Matthay MA. Intrapulmonary delivery of bone marrow-derived mesenchymal stem cells improves survival and attenuates endotoxin-induced acute lung injury in mice. J Immunol 2007;179:1855-63.
2. Kim ES, Chang YS, Choi SJ, Kim JK, Yoo HS, Ahn SY, et al. Intratracheal transplantation of human umbilical cord blood-derived mesenchymal stem cells attenuates Escherichia coli-induced acute lung injury in mice. Respir Res 2011;12:108.
3. Krasnodembskaya A, Song Y, Fang X, Gupta N, Serikov V, Lee JW, et al. Antibacterial effect of human mesenchymal stem cells is mediated in part from secretion of the antimicrobial peptide LL-37. Stem Cells 2010;28:2229-38.
4. Shin S, Kim Y, Jeong S, Hong S, Kim I, Lee W, et al. The therapeutic effect of human adult stem cells derived from adipose tissue in endotoxemic rat model. Int J Med Sci 2013;10:8-18.
5. Danchuk S, Ylostalo JH, Hossain F, Sorge R, Ramsey A, Bonvillain RW, et al. Human multipotent stromal cells attenuate lipopolysaccharide-induced acute lung injury in mice via secretion of tumor necrosis factor-alpha-induced protein 6. Stem Cell Res Ther 2011;2:27.
6. Lim R, Milton P, Murphy SV, Dickinson H, Chan ST, Jenkin G. Human mesenchymal stem cells reduce lung injury in immunocompromised mice but not in immunocompetent mice. Respiration 2013;85:332-41.
7. Li J, Li D, Liu X, Tang S, Wei F. Human umbilical cord mesenchymal stem cells reduce systemic inflammation and attenuate LPS-induced acute lung injury in rats. J Inflamm (Lond) 2012;9:33.
8. Zhao Y, Yang C, Wang H, Li H, Du J, Gu W, et al. Therapeutic effects of bone marrow-derived mesenchymal stem cells on pulmonary impact injury complicated with endotoxemia in rats. Int Immunopharmacol 2013;15:246-53.
9. Chang CL, Leu S, Sung HC, Zhen YY, Cho CL, Chen A, et al. Impact of apoptotic adipose-derived mesenchymal stem cells on attenuating organ damage and reducing mortality in rat sepsis syndrome induced by cecal puncture and ligation. J Transl Med 2012;10:244.
10. Yang B, Bai B, Liu CX, Wang SQ, Jiang X, Zhu CL, et al. Effect of umbilical cord mesenchymal stem cells on treatment of severe acute pancreatitis in rats. Cytotherapy 2013;15:154-62.
11. Yilmaz S, Inandiklioglu N, Yildizdas D, Subasi C, Acikalin A, Kuyucu Y, et al. Mesenchymal stem cell: does it work in an experimental model with acute respiratory distress syndrome? Stem Cell Rev 2013;9:80-92.
12. Huang Y, Yin W, Zhang XM, Wang YT, Yu HY, Hao L, et al. Optimal conditions of bone marrow mesenchymal stem cells on paraquat-induced acute lung injury in rats. Zhonghua Lao Dong Wei Sheng Zhi Ye Bing Za Zhi 2012;30:645-9.
13. Qin ZH, Xu JF, Qu JM, Zhang J, Sai Y, Chen CM, et al. Intrapleural delivery of MSCs attenuates acute lung injury by paracrine/endocrine mechanism. J Cell Mol Med 2012;16:2745-53.
14. Tai WL, Dong ZX, Zhang DD, Wang DH. Therapeutic effect of intravenous bone marrow-derived mesenchymal stem cell transplantation on early-stage LPS-induced acute lung injury in mice. Nan Fang Yi Ke Da Xue Xue Bao 2012;32:283-90.
15. Curley GF, Ansari B, Hayes M, Devaney J, Masterson C, Ryan A, et al. Effects of intratracheal mesenchymal stromal cell therapy during recovery and resolution after ventilator-induced lung injury. Anesthesiology 2013;118:924-32.
16. Hannoush EJ, Elhassan I, Sifri ZC, Mohr AA, Alzate WD, Livingston DH. Role of bone marrow and mesenchymal stem cells in healing after traumatic injury. Surgery 2013;153:44-51.
17. Ionescu L, Byrne RN, van Haaften T, Vadivel A, Alphonse RS, Rey-Parra GJ, et al. Stem cell conditioned medium improves acute lung injury in mice: in vivo evidence for stem cell paracrine action. Am J Physiol Lung Cell Mol Physiol 2012;303:L967-L977.
18. Xu YL, Liu YL, Wang Q, Li G, Lu XD, Kong B. Intravenous transplantation of mesenchymal stem cells attenuates oleic acid induced acute lung injury in rats. Chin Med J (Engl) 2012;125:2012-8.
19. Chen S, Chen L, Wu X, Lin J, Fang J, Chen X, et al. Ischemia postconditioning and mesenchymal stem cells engraftment synergistically attenuate ischemia reperfusion-induced lung injury in rats. J Surg Res 2012;178:81-91.
20. Wang L, Tu XH, Zhao P, Song JX, Zou ZD. Protective effect of transplanted bone marrow-derived mesenchymal stem cells on pancreatitis-associated lung injury in rats. Mol Med Report 2012;6:287-92.
21. Gupta N, Krasnodembskaya A, Kapetanaki M, Mouded M, Tan X, Serikov V, et al. Mesenchymal stem cells enhance survival and bacterial clearance in murine Escherichia coli pneumonia. Thorax 2012;67:533-9.
22. Curley GF, Hayes M, Ansari B, Shaw G, Ryan A, Barry F, et al. Mesenchymal stem cells enhance recovery and repair following ventilator-induced lung injury in the rat. Thorax 2012;67:496-501.
23. Krasnodembskaya A, Samarani G, Song Y, Zhuo H, Su X, Lee JW, et al. Human mesenchymal stem cells reduce mortality and bacteremia in gram-negative sepsis in mice in part by enhancing the phagocytic activity of blood monocytes. Am J Physiol Lung Cell Mol Physiol 2012;302:L1003-L1013.
24. Wu Q, Wang F, Hou Y, Chen S, Wang B, Lu F, et al. The effect of allogenetic bone marrow-derived mesenchymal stem cell transplantation on lung aquaporin-1 and -5 in a rat model of severe acute pancreatitis. Hepatogastroenterology 2012;59:965-76.
25. Song L, Xu J, Qu J, Sai Y, Chen C, Yu L, et al. A therapeutic role for mesenchymal stem cells in acute lung injury independent of hypoxia-induced mitogenic factor. J Cell Mol Med 2012;16:376-85.
26. Liang ZX, Sun JP, Wang P, Tian Q, Yang Z, Chen LA. Bone marrow-derived mesenchymal stem cells protect rats from endotoxin-induced acute lung injury. Chin Med J (Engl ) 2011;124:2715-22.
27. Sun CK, Yen CH, Lin YC, Tsai TH, Chang LT, Kao YH, et al. Autologous transplantation of adipose-derived mesenchymal stem cells markedly reduced acute ischemia-reperfusion lung injury in a rodent model. J Transl Med 2011;9:118.
28. Hannoush EJ, Sifri ZC, Elhassan IO, Mohr AM, Alzate WD, Offin M, et al. Impact of enhanced mobilization of bone marrow derived cells to site of injury. J Trauma 2011;71:283-9.
29. Sun J, Han ZB, Liao W, Yang SG, Yang Z, Yu J, et al. Intrapulmonary delivery of human umbilical cord mesenchymal stem cells attenuates acute lung injury by expanding CD4+CD25+ Forkhead Boxp3 (FOXP3)+ regulatory T cells and balancing anti- and pro-inflammatory factors. Cell Physiol Biochem 2011;27:587-96.
30. Zhu F, Guo GH, Huang S, Chen RS, Chen W, Zhan JH. The effect of bone marrow mesenchymal stem cells engraftment on lung tissue at early stage of smoke inhalation injury in rabbits. Zhongguo Wei Zhong Bing Ji Jiu Yi Xue 2011;23:18-20.
31. Zhu F, Guo GH, Chen W, Peng Y, Xing JJ, Wang NY. Effect of bone marrow-derived mesenchymal stem cells transplantation on the inflammatory response and lung injury in rabbit with inhalation injury. Zhonghua Shao Shang Za Zhi 2010;26:360-5.
32. Yagi H, Soto-Gutierrez A, Kitagawa Y, Tilles AW, Tompkins RG, Yarmush ML. Bone marrow mesenchymal stromal cells attenuate organ injury induced by LPS and burn. Cell Transplant 2010;19:823-30.
33. Mei SH, Haitsma JJ, Dos Santos CC, Deng Y, Lai PF, Slutsky AS, et al. Mesenchymal stem cells reduce inflammation while enhancing bacterial clearance and improving survival in sepsis. Am J Respir Crit Care Med 2010;182:1047-57.
34. Yagi H, Soto-Gutierrez A, Navarro-Alvarez N, Nahmias Y, Goldwasser Y, Kitagawa Y, et al. Reactive bone marrow stromal cells attenuate systemic inflammation via sTNFR1. Mol Ther 2010;18:1857-64.
35. Lee SH, Jang AS, Kim YE, Cha JY, Kim TH, Jung S, et al. Modulation of cytokine and nitric oxide by mesenchymal stem cell transfer in lung injury/fibrosis. Respir Res 2010;11:16.
36. Leblond AL, Naud P, Forest V, Gourden C, Sagan C, Romefort B, et al. Developing cell therapy techniques for respiratory disease: intratracheal delivery of genetically engineered stem cells in a murine model of airway injury. Hum Gene Ther 2009;20:1329-43.
37. Moodley Y, Atienza D, Manuelpillai U, Samuel CS, Tchongue J, Ilancheran S, et al. Human umbilical cord mesenchymal stem cells reduce fibrosis of bleomycin-induced lung injury. Am J Pathol 2009;175:303-13.
38. Gonzalez-Rey E, Anderson P, Gonzalez MA, Rico L, Buscher D, Delgado M. Human adult stem cells derived from adipose tissue protect against experimental colitis and sepsis. Gut 2009;58:929-39.
39. Nemeth K, Leelahavanichkul A, Yuen PS, Mayer B, Parmelee A, Doi K, et al. Bone marrow stromal cells attenuate sepsis via prostaglandin E(2)-dependent reprogramming of host macrophages to increase their interleukin-10 production. Nat Med 2009;15:42-9.
40. Zhao F, Zhang YF, Liu YG, Zhou JJ, Li ZK, Wu CG, et al. Therapeutic effects of bone marrow-derived mesenchymal stem cells engraftment on bleomycin-induced lung injury in rats. Transplant Proc 2008;40:1700-5.
41. Xu J, Qu J, Cao L, Sai Y, Chen C, He L, et al. Mesenchymal stem cell-based angiopoietin-1 gene therapy for acute lung injury induced by lipopolysaccharide in mice. J Pathol 2008;214:472-81.
42. Mei SH, McCarter SD, Deng Y, Parker CH, Liles WC, Stewart DJ. Prevention of LPS-induced acute lung inury in mice by mesenchymal stem cells overexpressing angiopoietin 1. PLoS Med 2007;4:e269.
43. Xu J, Woods CR, Mora AL, Joodi R, Brigham KL, Iyer S, et al. Prevention of endotoxin-induced systemic response by bone marrow-derived mesenchymal stem cells in mice. Am J Physiol Lung Cell Mol Physiol 2007;293:L131-L141.
44. Gao P, Yang X, Mungur L, Kampo S, Wen Q. Adipose tissue-derived stem cells attenuate acute lung injury through eNOS and eNOS-derived NO. Int J Mol Med 2013;31:1313-8.
45. Wu H, Li T, Xu J, Huang L. Effect of mesenchymal stem cells on expression of cyclophilin A in lipopolysaccharide-induced acute lung injury rat. Journal of International Pharmaceutical Research 2012;39:425-9.
46. Chen W, Zhu F, Guo GH, Zhan JH. Effect of bone marrow mesenchymal stem cells engraftment on secretion of inflammatory cytokine in the early stages of smoke inhalation injury in rabbits. Zhongguo Wei Zhong Bing Ji Jiu Yi Xue 2011;23:21-3.
47. Zhang F, Cheng J, Chu D-L, Sun Y-N, Wang C-L, Huang J. Prevention of bone marrow mesenchymal stem cell transplantation against acute lung injury in rabbits. Journal of Clinical Rehabilitative Tissue Engineering Research 2009;13:5225-8.
48. Zhao F, Li S-Q, Ti X-Y, Song L-Q, Li Z-K, Wu C-G, et al. Effects of bone marrow mesenchymal stem cells on transforming growth factor beta and monocyte chemoattractant protein-1 in lung injury rats. Journal of Clinical Rehabilitative Tissue Engineering Research 2008;12:5627-30.
49. Zhang S, Danchuk SD, Imhof KM, Semon JA, Scruggs BA, Bonvillain RW, et al. Comparison of the therapeutic effects of human and mouse adipose-derived stem cells in a murine model of lipopolysaccharide-induced acute lung injury. Stem Cell Res Ther 2013;4:13.
50. Chien MH, Bien MY, Ku CC, Chang YC, Pao HY, Yang YL, et al. Systemic human orbital fat-derived stem/stromal cell transplantation ameliorates acute inflammation in lipopolysaccharide-induced acute lung injury. Crit Care Med 2012;40:1245-53.
51. Bi LK, Tang B, Zhu B, Xie C-L, Li S, Lin T-X et al. Systemic delivery of IL-10 by bone marrow derived stromal cells has therapeutic benefits in sepsis therapy. Progress in Biochemistry and Biophysics 37[6], 678-685. 2010.
52. Yang H, Wen Y, Hou-You Y, Yu-Tong W, Chuan-ming L, Jian X, et al. Combined treatment with bone marrow mesenchymal stem cells and methylprednisolone in paraquat-induced acute lung injury. BMC Emerg Med 2013;13 Suppl 1:S5.
53. Maron-Gutierrez T, Silva JD, Asensi KD, Bakker-Abreu I, Shan Y, Diaz BL, et al. Effects of mesenchymal stem cell therapy on the time course of pulmonary remodeling depend on the etiology of lung injury in mice. Crit Care Med 2013;41:e319-e333.
54. Zhu F, Guo GH, Chen W, Wang NY. Effects of bone marrow-derived mesenchymal stem cells engraftment on vascular endothelial cell growth factor in lung tissue and plasma at early stage of smoke inhalation injury. World J Emerg Med 2010;1:224-8.
