# Supplementary material for: Efficacy of Mesenchymal Stromal Cell Therapy for Acute Lung Injury in Preclinical Animal Models: A Systematic Review
Source: PLoS One. 2016 Jan 28;11(1):e0147170. doi: 10.1371/journal.pone.0147170 (PMC4731557; doi:10.1371/journal.pone.0147170)
Supplement: S1 Table — (DOCX) [file pone.0147170.s002.docx]

**S1 Table**: General characteristics of all included studies

| **Author (year);**  **Sample size;**  **Country** | **Animal Model, Gender** | **ALI Model** | **Control Group; Resuscitation** | **MSC Source, Type; Fresh/**  **Frozen** | **MSC Dose, Delivery Time (h), and method of delivery** | **Control, Amount, Time** | **Outcomes** |
| --- | --- | --- | --- | --- | --- | --- | --- |
| Gupta (2007)A^7^;  N=NR in methods;  United States | C57BL/6 mouse, Male | Direct infection/inflammation: IT LPS | Disease; None | Syngeneic, Bone Marrow; Unclear | 7.5x10^5^, 4, IT | PBS, 30 µl, 4 h | Extra vascular lung water; BALF total protein; BALF cytokines (Cxcl2, TNFα); Lung MPO activity; BALF cytokines (IL10); Mortality |
| Gupta (2007)B^7^;  N=NR In methods;  United States | C57BL/6 mouse, Male | Direct infection/inflammation: IT LPS | Disease; None | Syngeneic, Bone marrow; Unclear | 7.5x10^5^, 4, IT | PBS, 30 µl, 4 h | Mortality |
| Mei (2007)A^50^;  N=30 (10 in groups we used);  Canada | C57BL/6J mouse, Female | Direct infection/inflammation: IT LPS | Disease, Sham; None | Syngeneic, Bone marrow; Fresh# | 2.5x10^5^, 0.5, IV | NS, unclear (assumed 100 µl), 0.5 h | BALF total protein; BALF albumin; BALF IgM; BALF neutrophils; BALF cytokines (IFN-γ, TNFα, IL6, IL1β, Cxcl2, JE, KC); Lung tissue cytokines (TNFα, IL6, Cxcl2, JE, KC) |
| Mei (2007)B^50^;  N=39 (19 in the groups we used);  Canada | C57BL/6J mouse, Female | Direct infection/inflammation: IT LPS | Disease, Sham; None | Syngeneic, Bone marrow; Fresh# | 2.5x10^5^, 0.5, IV | NS, unclear (assumed 100 µl), 0.5 h | Standardized lung histology score; Thickened alveolar wall |
| Xu (2007)^51^;  N=NR in methods;  United States | C57BL/6 mouse, Female | Indirect infection/inflammation: IP LPS | Disease, Sham; None | Syngeneic, Bone marrow; Unclear | 5.0x10^5^, 1, IV | Two arm:  a) Cell (fibroblast), 5x10^5^, 1 h  b) Nothing | Thickened alveolar wall; Neutrophils in interstitial space; Lung wet/dry weight ratio; BALF cytokines (IFN-γ, KC, IL1β, IL10) |
| Xu (2008)^49^;  N=NR in methods;  China | C57BL/6 mouse, Male | Direct infection/inflammation: LPS by nebulisation | Disease; None | Syngeneic, Bone marrow; Unclear | Not reported, 2, IV | NS, not reported, 2 h | Standardized lung histology score; Lung wet/dry weight ratio; BALF total protein; BALF neutrophils; Lung MPO activity; BALF cytokines (TNFα, IL10); Lung tissue mRNA cytokines (TNFα) |
| Zhao (2008) #1^56^;  N=20 (10 in the groups we used);  China | Sprague- Dawley rat, Female | Direct chemical injury: IT bleomycin | Diseased, Normal control; None | Syngeneic, Bone marrow; Unclear | 5.0x10^5^, 12, IV | DMEM-F12, 0.5 mL, 12 h | BALF cytokines (MCP-1) |
| Zhao (2008) #2^48^;  N=20 (10 in groups we used);  China | Sprague-Dawley rat, Not reported | Direct chemical injury: IT bleomycin | Disease, Sham; None | Syngeneic, Bone marrow; Unclear | 5.0x10^6^, 12, IV | Vehicle (assumed DMEM/F12), not reported, not reported (assumed 12 h) | Thickened alveolar wall |
| Gonzalez-Rey (2009)A^46^;  N=NR in methods;  Spain | BALb/C mouse, Not reported | Indirect infection/inflammation: CLP  (1 X 22 G) | Disease; None | Xenogenic, Adipose; Unclear  Two arm for survival:  a) Xenogenic, Adipose  b) Allogeneic, Adipose | 1.0x10^6^, 4, IP | Other (medium, DMEM), unclear, 4 h | Lung MPO activity; Bacterial clearance (peritoneum, blood, spleen and liver); Mortality |
| Gonzalez-Rey (2009)B^46^;  N=NR in methods;  Spain | BALb/C mouse, Not reported | Indirect infection/inflammation: IP LPS | Disease; None | Xenogenic, Adipose; Unclear | 1.0 x10^6^, 0.5, IP  Two arm for survival:  a) 1.0x10^6^  b) 3.0x10^5^ | Other (medium, DMEM), unclear, 0.5 h | Lung MPO activity; Lung tissue cytokines (TNFα, IL6, IL1β, Cxcl2, IL10, IFN-γ); Mortality |
| Leblond (2009)^44^;  N=NR in methods;  France | Swiss mouse, Male | Direct Chemical injury: IT 2% polidocanol | Disease, Sham; None | Allogeneic, Bone marrow; Unclear | 1.0x10^6^, 24, IT | Two arm:  a) PBS, unclear (assumed 25 µl), 24 h  b) Nothing | Mortality |
| Moodley (2009)^45^;  N=120 (72 in the groups we used);  Australia | SCID mouse, Not reported | Direct chemical injury: IN bleomycin | Disease, Sham; None | Xenogenic, Umbilical cord; Unclear | 1.0x10^6^, 24, IV | Two arm:  a) NS, 0.2 ml, 24 h  b) Cell fibroblast), 1x10^6^, 24 h | Neutrophils in interstitial space; Thickened alveolar wall; Lung tissue mRNA cytokines (IL10, IFN-γ, IL6, TNFα) |
| Nemeth (2009)^47^;  N=N values only reported on an outcome basis in supplementary methods;  United States | C57BL/6 mouse, Male | Indirect infection/inflammation: CLP  (2 X 21 G) | Disease, Sham; Fluids and antibiotics | Allogeneic, Bone marrow; Unclear  Four arm:  a) FVB BMSC  b) BALB/c BMSC  c) B6 BMSC  d) B6 BMSC | Four arm:  a) 1x10^6^, 0  b) 1x10^6^, 0  c) 1x10^6^, 0  d) 1x10^6^, 1  IV | Two arm:  a) PBS, 0.3 ml, not reported (assumed 0 h)  b) Cells (fibroblast), not reported (assumed 1x10^6^), not reported (assumed 0 h) | Mortality |
| Zhang (2009)^55^;  N=18 (12 in the groups we used);  China | Rabbit, Not reported | Direct infection/inflammation: IT LPS | Diseased, Sham; None | Allogeneic, Bone marrow; Unclear | 1x10^5^, 0.5, IV | Saline, 2 mL, 0.5 h | Lung wet/dry ratio; neutrophils in interstitial space; BALF total protein |
| Bi (2010)^59^;  N=40 (20 in the groups we used);  China | C57BL/6 mouse, Not reported | Indirect infection/inflammation: CLP  (2 X 21 G) | Disease, Sham; Fluid | Xenogenic, Bone marrow; Unclear | 1.0x10^6^, 1, IV | PBS, 1 ml, 1 h | Thickened alveolar wall; Lung tissue mRNA cytokines (IL6, TNFα); Mortality |
| Krasnodembskaya (2010)^11^;  N=NR in methods;  United States | C57BL/6 mouse, Male | Direct infection/inflammation: IT bacteria (*E.coli*) | Disease; None | Xenogenic, Bone marrow; Fresh# | 1.0x10^6^, 4, IT | PBS, unclear (assumed 30 ul), 4 h | BALF total protein; BALF cytokines (Cxcl2); BALF neutrophils; Bacterial clearance (BALF and lung homogenates) |
| Lee (2010)^43^;  N=NR in methods;  Korea | Sprague-Dawley rat, Female | Direct chemical injury: Inhalation of bleomycin | Disease, Sham; None | Syngeneic, Bone marrow; Unclear | 1.0x10^6^, 96, IV | Unclear (believed to be nothing) | Neutrophils in interstitial space, Lung wet/dry weight ratio, BALF neutrophils; BALF cytokines (IL6, TNFα, IL1β), Mortality |
| Mei (2010)A^41^;  N= NR in methods;  Canada | C57Bl/6J mouse, Female | Indirect infection/inflammation: CLP (1 X 22 G) | Disease, Sham; Fluid | Syngeneic, Bone Marrow; Fresh# | 2.5x10^5^, 6, IV | NS, 100 µl, 6 h | Neutrophils in interstitial space; Extravascular lung water; BALF total protein; BALF albumin; BALF cytokines (IL6, IL1β, IL10, KC, and JE); Bacterial clearance; Mortality |
| Mei (2010)B^41^;  N= NR in methods;  Canada | C57Bl/6J mouse, Female | Indirect infection/inflammation: CLP (1 X18 G) | Disease, Sham; Fluid and antimicrobial therapy | Syngeneic, Bone Marrow; Fresh# | 2.5x10^5^, 6, IV | NS, 100 µl, 6 h | Mortality |
| Yagi (2010)#1^40^;  N=NR in methods;  United States | Sprague-Dawley rat, Male | Indirect infection/inflammation: IP LPS | Disease; None | Xenogenic, Bone marrow; Fresh# | 2.0x10^6^, 0, IM | Nothing | Standardized lung histology score |
| Yagi (2010)#2^42^;  N=28 (14 in the groups we used);  United States | Sprague-Dawley rat, Male | Indirect infection/inflammation: IP LPS | Disease, Sham; None | Xenogenic, Bone marrow; Fresh# | 2.0x10^6^, 0, IM | PBS, unclear (assumed 500 µl), unclear (assumed 0 h) | Neutrophils in interstitial space |
| Zhu (2010) #1^62^;  N=64;  China | New Zealand white rabbit, Not reported | Trauma: Smoke | Disease; None | Syngeneic, Bone marrow; Unclear | 1.0x10^7^, 0, IV | PBS, 10 ml, 0 h | Lung wet/dry weight ratio |
| Zhu ( 2010) #2^39^;  N=16;  China | New Zealand big ear rabbits, Male | Trauma: Smoke | Disease, Normal control; None | Syngeneic, Bone marrow; Unclear | 1x10^7^, 0, IV | PBS, 10 mL, unclear | Standardized lung histology score; Extra vascular lung water |
| Chen (2011)^54^;  N=56 (48 in the groups we used);  China | New Zealand rabbits, Mixed | Trauma: Smoke | Diseased, Sham; None | Syngeneic, Bone marrow; Unclear | 1x10^7^, 0, IV | PBS, 10 mL, 0 h | Lung tissue cytokine (TNF-α, IL1β, IL10, IL6) |
| Danchuk (2011)A^13^;  N= NR in methods;  United States | BALB/C mouse, Female | Direct infection/inflammation: OA LPS | Disease, Sham; None | Xenogenic, Bone marrow; Fresh# | Two doses of 2.5x10^5^ cells = 5x10^5^ total cells, 4 and 4.5, OA | Two arm:  a) PBS, total of 400 μl; 4 and 4.5 h  b) Cell (fibroblast); total of 5x10^5^ cells; 4 and 4.5 h | Thickened alveolar wall; Neutrophils in interstitial space; Standardized lung histology score; BALF total protein; BALF neutrophils; BALF cytokines (IL1β, IL6, JE, and Cxcl2); Lung MPO activity |
| Danchuk (2011)B^13^;  N= NR in methods;  United States | BALB/C mouse, Female | Direct infection/inflammation: OA LPS | Disease, Sham; None | Xenogenic, Bone marrow; Fresh# | Two doses of 2.5x10^5^ cells = 5x10^5^ total cells, 4 and 4.5, OA | Two arm:  a) PBS, total of 400 μl; 4 and 4.5 h  b) Cell (fibroblast); total of 5x10^5^ cells; 4 and 4.5 h | Lung wet/dry ratio |
| Danchuk (2011)C^13^;  N=NR in methods;  United States | BALB/C mouse, Female | Direct infection/inflammation: OA LPS | Disease, Sham; None | Xenogenic, Bone marrow; Fresh# | 5x10^5^, 4, Three arm:  a) OA  b) IV  c) IP | Two arm:  a) PBS, unclear (assumed to be 400 μl), unclear (assumed to be 4 h)  b) Cell (fibroblast); unclear (assumed to be 5x10^5^ cells), unclear (assumed to be 4 h) | BALF total protein |
| Hannoush (2011)^36^;  N=30 (12 in the groups we used)‡;  United States | Sprague-Dawley rat, Male | Trauma: LC | Disease, Sham; None | Allogeneic, Bone marrow; Unclear | 5.0x10^6^, 0, IV | NS, unclear, unclear (assumed to be 0 h) | Standardized lung histology score |
| Kim (2011)^10^;  N=344 (287 in the groups we used);  Korea | ICR mouse, Male | Direct infection/inflammation: IT bacteria (*E. coli*) | Disease, Sham; Antibiotics | Xenogenic, Umbilical cord; Unclear | 1.0x10^5^, 3, IT | Two arm:  a) PBS, 0.05 ml, 3 h  b) Cell (fibroblast ), 1x10^5^ cells, 3 h | Neutrophils in interstitial space; Thickened alveolar wall; Lung wet/dry ratio; Lung MPO activity; Lung tissue cytokines (TNFα, IL1β, IL6, Cxcl2); Bacterial clearance (BALF and blood); Lung tissue cytokines (IL1β, JE, Cxcl2, TNFα); Mortality |
| Liang (2011)^34^;  N=65 (50 were in the groups we used);  China | Wistar rat , Female | Indirect infection/inflammation: IV LPS | Disease; None | Syngeneic, Bone marrow; Unclear | 1.0x10^6^, 2, IV | NS, 0.4 ml, 2 h | Thickened alveolar wall; Lung wet/dry weight ratio; BALF total protein; BALF neutrophils; Lung MPO activity; Mortality |
| Sun (2011) #1^37^;  N=Unclear (possible N=80);  China | BALb/C mouse, Male | Direct infection/inflammation: IT LPS | Disease; None | Xenogenic, Umbilical cord; Unclear | 1.0x10^6^, 4, IT | PBS, not reported, 4 h | Neutrophils in interstitial space; Extra vascular lung water, BALF total protein, BALF cytokines (TNFα, IL10, Cxcl2, IFN-γ), Mortality |
| Sun (2011) #2^35^;  N=24 (16 in the groups that we used);  Taiwan | Sprague-Dawley rat, Male | Pulmonary ischemia/ reperfusion | Disease, Sham; None | Autologous, Adipose; Fresh | 3 doses: 1.5x10^6^*, 1 and 6 and 24, IV | Other (medium, DMEM), not reported, not reported | Thickened alveolar wall; Hypoxemia; Lung tissue cytokines (TNFα); Lung tissue mRNA cytokines (IL1β, TNFα, IL10) |
| Zhu ( 2011)^38^;  N=48;  China | New Zealand big ear rabbits, Not reported | Trauma: Smoke | Disease; None | Syngeneic, Bone marrow; Unclear | 1x10^7^, 0, IV | PBS, 10 ml, unclear | Standardized lung histology score |
| Chang (2012)^17^;  N=64 (32 in the groups we used);  China | Sprague-Dawley rat, Male | Indirect infection/inflammation: CLP  (2 X 18 G) | Disease, Sham; None | fAutologous, Adipose; Fresh | 3 doses of 1.2x10^6^ = total 3.6 x 10^7^, 0.5 and 6 and 18, IP | NS, three doses of 3.0 cc = total 9.0 cc, 0.5, 6 and 18 h | Mortality; Lung wet/dry weight ratio; Hypoxemia |
| Chen (2012)^27^;  N=75 (30 in the groups we used);  China | Sprague-Dawley rat, Male | Pulmonary ischemia/ reperfusion | Disease, Sham; None | Syngeneic, Bone marrow; Unclear | 1.0x10^6^, 0, IV | Nothing | Standardized lung histology score; Neutrophils in interstitial space; Extra vascular lung water; Lung wet/dry weight ratio; Hypoxemia; Lung MPO activity; Lung tissue mRNA cytokines (TNFα, IL10) |
| Chien (2012)^58^;  N=Unclear (possible N≥20 (10in groups we used));  Taiwan | BALB/c mouse, Male | Direct infection/inflammation: IT LPS | Disease, Sham; None | Xenogenic, Adipose; Unclear | 3.0x10^5^, 0.33 (20 min), IV | PBS, not reported (assumed 50 µl), not reported (assumed 20 min) | Standardized lung histology score; Neutrophils in interstitial space; Thickened alveolar wall; BALF total protein; Lung wet/dry weight ratio; BALF neutrophils; Lung tissue cytokines (TNFα) |
| Curley (2012)A^30^;  N=NR in methods;  Ireland | Sprague-Dawley rat, Male | Ventilator-induced | Disease; None | Syngeneic, Bone Marrow; Unclear | Two doses of 2.0x10^6^ cells = 4x10^6^ total cells, 0 and 24, IV | PBS, total 1000 µl, 0 and 24 h | Thickened alveolar wall; Lung wet/dry ratio; BALF total protein; Alveolar-arterial oxygen difference; BALF neutrophils; BALF cytokines (TNFα, IL6, IL10) |
| Curley (2012)B^30^;  N= NR in methods;  Ireland | Sprague-Dawley rat, Male | Ventilator-induced | Disease; None | Syngeneic, Bone marrow; Unclear | Two doses of 2.0x10^6^ cells = 4x10^6^ total cells, 0 and 24, IV | Two arm:  a) PBS, total 1000 µl, 0 and 24 h  b) Cells (fibroblast), total 4x10^6^, 0 and 24 h | BALF neutrophils; BALF cytokines (TNFα, IL6) |
| Curley (2012)C (companion thesis);  N=NR in methods;  Ireland | Sprague-Dawley rat, Male | Ventilator-induced | Disease; None | Syngeneic, Bone marrow; Unclear | Two doses of 2.0x10^6^ cells = 4x10^6^ total cells, 0 and 24, IV | PBS, total 1000 µl, 0 and 24 h | BALF neutrophils; BALF cytokines (TNFα, IL6) |
| Gupta (2012)^29^;  N= NR in methods;  United States | C57BL/6 mouse, Male | Direct infection/inflammation: IT bacteria (*E. coli* K1) | Disease; None | Syngeneic, Bone marrow; Unclear | 7.5x10^5^, 4, IT | Two arm:  a) PBS, 30 µl, 4 h  b) Cell (fibroblast), 7.5x10^5^, 4 h | Extra vascular lung water; Lung MPO activity; BALF cytokines (TNFα, Cxcl2, IL10); Bacterial clearance; Mortality |
| Huang (2012)^20^;  N=215 (135 in the groups we used)#;  China | Sprague-Dawley rat, Mixed | Indirect chemical injury: IP Paraquat | Diseased, Sham; None | Syngeneic, Bone marrow; Unclear | Eight arm:  a) 1x10^5^, 6  b) 1x10^6^, 6  c) 1x10^7^, 6  d) 1x10^8^, 6  e) 1x10^7^, 1  f) 1x10^7^, 6  g) 1x10^7^, 12  h) 1x10^7^, 24  IV | Nothing | Lung wet/dry ratio |
| Ionescu (2012)^25^;  N=Unclear (possible N≥35 (20 in the groups we used));  Canada | C57BL/6 mouse, Male | Direct infection/inflammation: IT LPS | Disease, Sham; None | Syngeneic, Bone marrow; Unclear | 2.5x10^5,^ 4, IT | Three arms:  a) Vehicle (DMEM), 30 µl, 4 h  b) Cell (fibroblast), 2.5x10^5^ cells, 4 h  c) Other (FCM), 30 µl, 4 h | Standardized lung histology score; Thickened alveolar wall; Proteinaceous debris; Lung wet/dry weight ratio; BALF neutrophils |
| Krasnodembskaya (2012)A^31^;  N=NR in methods;  United States and /or Italy | C57BL/6J mouse, Male | Indirect infection/inflammation: IP bacteria (*P. aeruginosa)* | Disease; None | Xenogenic, Bone marrow; Fresh# | 1.0x10^6^, 1, IV | Two arm:  a) PBS, 150 µl, 1 h  b) Cell (fibroblast), 1x10^6^, 1 h | Bacterial clearance |
| Krasnodembskaya (2012)B^31^;  N=NR in methods;  United States and /or Italy | C57BL/6J mouse, Male | Indirect infection/inflammation: IP bacteria (*P. aeruginosa)* | Disease; None | Xenogenic, Bone marrow; Fresh# | 1.0x10^6^, 1, IV | Two arm:  a) PBS, 150 µl, 1 h  b) Cell (fibroblast), 1x10^6^, 1 h | Mortality |
| Li (2012)A^15^;  N=60 (45 in the groups we used);  China | Sprague–Dawley rat, Male | Indirect infection/inflammation: IP LPS | Disease, Sham; None | Xenogenic, Umbilical cord; Unclear | 5x10^5^, 1, IV | NS, 300 μl, 1 h | Lung wet/dry ratio; BALF total protein; Lung MPO activity; BALF neutrophils; Neutrophils in interstitial space; Thickened alveolar wall |
| Li (2012)B^15^;  N=80 (60 in the groups we used);  China | Sprague–Dawley rat, Male | Indirect infection/inflammation: IP LPS | Disease, Sham; None | Xenogenic, Umbilical cord; Unclear | 5x10^5^, 1, IV | Two arm:  a) NS, 300 μl, 1 h  b) Cell (fibroblast), 5×10^5^ cells, 1 h | Standardized lung histology score; Mortality |
| Qin (2012)^21^;  N=42 (36 in groups we used);  China | Sprague-Dawley rat, Male | Direct infection/inflammation: IT LPS | Disease; None | Syngeneic, Bone Marrow; Unclear | 1.0x10^6^, 0, IPL | PBS, 300 µl, unclear | Standardized lung histology score; BALF total protein; Lung wet/dry weight ratio; BALF cytokines (TNFα, IL10); Lung tissue cytokines (TNFα, IL10); Lung tissue mRNA cytokines (TNFα, IL10); BALF neutrophils; Lung MPO activity |
| Song (2012)^33^;  N=64‡;  China | BALB/c mouse, Not reported | Direct infection/inflammation: IN LPS | Disease; None | Syngeneic, Bone marrow; Unclear | 5.0x10^5^/  mL, Unclear (assumed to be 0), IV | PBS, unclear, unclear (assumed 0 h) | Standardized lung histology score; Thickened alveolar wall; Lung wet/dry weight ratio; BALF total protein; Lung MPO activity; Lung tissue cytokines (TNFα, IL10); Lung tissue mRNA cytokines (TNFα, IL10); Neutrophils in interstitial space; Extra vascular lung water |
| Tai (2012)^22^;  N=36 (24 in the groups we used);  China | Kunming mouse, Not reported | Direct infection/inflammation: IT LPS | Disease, Sham; None | Syngeneic, Bone marrow; Unclear | 5.0x10^6^, 1, IV | PBS, 100 µl, 1 h | Lung wet/dry weight ratio; BALF total protein; BALF cytokines (TNFα, IL6, IL10); BALF neutrophils; Lung MPO activity |
| Wang (2012)^28^;  N=75 (50 in the groups we used);  China | Sprague-Dawley rat, Male | Indirect chemical injury: SAP by 5% sodium taurocholate | Disease, Sham; None | Syngeneic, Bone marrow; Unclear | 1.0x10^6^, 2, IV | Other (medium, DMEM), 1 mL, 2 h | Thickened alveolar wall; Lung wet/dry weight ratio; Lung MPO activity; Lung tissue mRNA cytokines (TNFα) |
| Wu (2012)#1A^32^;  N=20;  China | Sprague-Dawley rat, Male | Indirect infection/inflammation: SAP by 5% sodium taurocholate | Disease; Fluid | Syngeneic, Bone marrow; Unclear | ~5.0x10^5^ – 1.0x10^6^/  mL (10mL/kg), 1, IV | Vehicle (L-DMEM), 10 mL/kg, 1 h | Standardized lung histology score; Lung wet/dry weight ratio |
| Wu (2012)#1B^32^;  N=20;  China | Sprague-Dawley rat, Male | Indirect infection/inflammation: SAP by 5% sodium taurocholate | Disease; Fluid | Syngeneic, Bone marrow; Unclear | Two arm:  a) one dose of ~5.0x10^5^ – 1.0x10^6^/  mL (10mL/kg), 1  b) two doses of ~5.0x10^5^ – 1.0x10^6^/  mL (10mL/kg), 1 and 7  IV | Vehicle (L-DMEM), 10 mL/kg, two arm: a)1 hour b) 1 and 7 hours | Standardized lung histology score; Lung wet/dry weight ratio |
| Wu (2012)#1C^32^;  N=40;  China | Sprague-Dawley rat, Male | Indirect infection/inflammation: SAP by 5% sodium taurocholate | Disease; Fluid | Syngeneic, Bone marrow; Unclear | Unclear, believed to be  two arm:  a) one dose of ~5.0x10^5^ – 1.0x10^6^/  mL (10mL/kg), 1  b) two doses of ~5.0x10^5^ – 1.0x10^6^/  mL (10mL/kg), 1 and 7  IV | Vehicle (L-DMEM), 10 mL/kg, two arm: a)1 hour b) 1 and 7 hours | Mortality; Extra vascular lung water |
| Wu (2012) #2^53^;  N=90 (54 in the groups we used);  China | Sprague-Dawley rats, Male | Indirect infection/inflammation: IV LPS | Diseased, Sham; None | Xenogenic, Bone marrow; Unclear | Two arm:  a) 1x10^6^,  b) 5x10^5^, Unclear, IV | Nothing | Lung MPO, Lung tissue cytokines (TNFα, IL1β) |
| Xu (2012)^26^;  N=42;  China | Sprague–Dawley rat, Male | Indirect chemical injury: IV Oleic Acid | Disease; None | Syngeneic, Bone Marrow; Unclear | 2.0x10^6^, 0.17, IV | PBS, 60 μl,10 min | Standardized lung histology score; Lung wet/dry weight ratio; Lung tissue cytokines (TNFα, IL10, KC); Neutrophils in interstitial space |
| Curley (2013)^23^;  N=40 (32 within the groups that we used);  Ireland | Sprague–Dawley rat, Male | Ventilator-induced | Disease; None | Syngeneic, Bone marrow; Unclear | 4.0x10^6^, 0.25-0.5, Two arm: 1)IT  2)IV | Three arm:  a) Nothing  b)PBS, 300 µl, unclear (assumed to be 0.25-0.5 h)  c) Cell (fibroblast), 4×10^6^, unclear (assumed to be 0.25-0.5 h) | - Thickened alveolar wall; Lung wet/dry weight ratio; BALF total protein; Hypoxemia; Alveolar-arterial oxygen difference; BALF neutrophils; BALF cytokines (TNFα, IL6, IL10) |
| Gao (2013)^52^;  N=25 (20 in the groups we used);  China | Sprague-Dawley rat, Not reported | Indirect infection/inflammation: IP LPS | Disease, Sham; None | Xenogenic, Adipose; Unclear | 5.0x10^5^, Not reported, IV | NS, not reported, not reported | Lung wet/dry weight ratio |
|  |  |  |  |  |  |  |  |
| Hannoush (2013)^24^;  N=36 (30 in the groups we used)‡;  United States | Sprague–Dawley rat, Male | Trauma: Mesenteric LDL + LC | Disease; None | Syngeneic, Bone Marrow; Unclear | 5.0x10^6^, 0.08, IV | Four arm:  a) Nothing (following LC)  b) Nothing (following LC + laparotomy without lymph duct ligation)  c) Nothing (following LC + lymph duct ligation)  d) Cells (BMDC), 1x10^8^, not reported | Standardized lung histology score |
| Lim (2013)A^14^;  N=30 (unclear number in groups we used);  Australia | SCID mouse, Female | Direct chemical injury: IN bleomycin | Disease, Sham; None | Xenogenic, Bone marrow; Fresh# | 1x10^6^, 24, IV | NS, not reported, 24 h | Lung wet/dry ratio; Lung tissue mRNA cytokines (TNFα) |
| Lim (2013)B^14^;  N=30 (unclear number in groups we used);  Australia | C57Bl/6 mouse, Female | Direct chemical injury: IN bleomycin | Disease, Sham; None | Xenogenic, Bone marrow; Fresh# | 1x10^6,^ 24, IV | NS, not reported, 24 h | Lung wet/dry ratio; Lung tissue mRNA cytokines (TNFα) |
| Maron-Gutierrez (2013)A^61^;  N=Unclear (143 total animals used in A and B, appears that N=36 in the groups we used);  Canada and Brazil | C57BL/6 mouse, Female | Direct infection/inflammation: IT LPS | Disease, Sham; None | Syngeneic, Bone marrow; Unclear | 1.0x10^5^, 24, IV | NS, 0.05 ml, 24 h | Neutrophils in interstitial space; Lung tissue cytokines (IL1β, IL6, IL10, IFN-γ, JE, KC); BALF neutrophils |
| Maron-Gutierrez (2013)B^61^;  N=Unclear (143 total animals used in A and B, appears that N=36 in the groups we used);  Canada and Brazil | C57BL/6 mouse, Female | Indirect infection/inflammation: IP LPS | Disease, Sham; None | Syngeneic, Bone marrow; Unclear | 1.0x10^5^, 24, IV | NS, 0.05 ml, 24 h | Neutrophils in interstitial space; Lung tissue cytokines (IL1β, IL6, IL10, IFN-γ, JE, KC); BALF neutrophils |
| Shin (2013)^12^;  N=15 (10 in the groups we used);  Korea | Sprague–Dawley rat, Male | Indirect infection/inflammation: IV LPS | Disease, Sham; None | Xenogenic, Adipose; Unclear | 2x10^6^, 0.5, IV | NS, 100  µl, 0.5 h | Lung tissue cytokines (TNFα, IL6, IL10); Thickened alveolar wall; Neutrophils in interstitial space; Standardized lung histology score |
| Yang (2013) #1A^18^;  N=72 (60 in the groups we used);  China | Sprague-Dawley rat, Male | Indirect chemical injury: SAP by 5% sodium taurocholate | Disease, Sham; None | Xenogenic, Umbilical cord; Frozen | 5.0x10^6^, Four arm:  1) 0  2) 1  3) 6  4) 12  IV | NS, unclear, unclear (either 0 or 1 h) | Standardized lung histology score; Mortality |
| Yang (2013) #1B^18^;  N=72 (60 in the groups we used);  China | Sprague-Dawley rat, Male | Indirect chemical injury: SAP by 5% sodium taurocholate | Disease, Sham; None | Xenogenic, Umbilical cord; Frozen | Four arm:  1) 1x10^7^  2) 5x10^6^  3) 5x10^5^  4) 5x10^4^  1, IV | NS, unclear, unclear (either 0 or 1 h) | Neutrophils in interstitial space; Standardized lung histology score; Mortality; Extra vascular lung water |
| Yang (2013) #1C^18^;  N=36 (24 in groups we used);  China | Sprague-Dawley rat, Male | Indirect chemical injury: SAP by 5% sodium taurocholate | Disease, Sham; None | Xenogenic, Umbilical cord; Frozen | 5.0x10^6^, 0, IV | NS, unclear, unclear (assumed 0 h) | Neutrophils in interstitial space |
| Yang (2013) #2^60^;  N=102 (48 in the groups we used);  China | Sprague-Dawley rat, Female | Indirect chemical injury: IP Paraquat | Disease, Sham; None | Allogeneic, Bone marrow; Unclear | 1.0x10^7^, 6, IV | Nothing | Lung wet/dry weight ratio; Mortality |
| Yilmaz (2013)^19^;  N=18;  Turkey | Wistar albino rat, Male | Ventilator-induced | Disease; None | Syngeneic, Bone marrow; Unclear | 7.5x10^5^, 2, IT | Nothing | Hyaline membrane; Extra vascular lung water; Neutrophils in interstitial space; Lung MPO activity; Lung tissue cytokines (IL1β, IL6, Cxcl2) |
| Zhang (2013)^57^;  N=NR in methods;  United States | C57Bl/6 mouse, Female | Direct infection/inflammation: OA LPS | Disease, Sham; None | Two arm:  a) Xenogenic, Adipose  Fresh#  b) Syngeneic, Adipose;  Fresh# | Two doses of 3.75x10^5^ cells = 7.5x10^5^ total cells, 4 and 4.5, OA | Vehicle (HBSS), total of 150 µl, 4 h | Thickened alveolar wall; BALF total protein; BALF albumin; BALF neutrophils; Lung MPO activity; Lung tissue cytokines (Cxcl2, IL1β, IFN-γ, IL10); Lung tissue mRNA cytokines (IL1β, TNFα, IL10) |
| Zhao (2013)^16^;  N=102 (51 in the groups we used);  China | Sprague–Dawley rat, Female | Combination: IV LPS + LC | Disease; None | Syngeneic, Bone Marrow; Unclear | 2.5x10^6^, 2, IV | NS, 0.5 mL, unclear (assumed 2 h) | Standardized lung histology score; Hypoxemia; BALF cytokines (TNFα, IL1β, IL6, IL10); BALF neutrophils; Mortality |

* Unclear if this is a total amount or individual dose

‡N value was a range was provided in the methods and the highest number was used to calculate the N value

#Number vary from those presented by Huang 2012, as number do not add up correctly in publication

Abbreviations: BALFF = Bronchoalveolar lavage fluid; CLP = Cecal ligation and puncture; DMEM = Dulbecco’s Modified Eagle’s Medium; FCM=Fibroblast conditioned media; G= Gauge; h = hour; IM = Intramuscular; IN = Intranasal; IP = Intraperitoneal; IPL = Intrapleural; IT = Intratracheal; IV = Intravenous; LC = Lung Contusion; LDL = Lymph Duct Ligation; LPS = Lipopolysaccharide; MPO = Myeloperoxidase; NR = Not reported; NS = Normal Saline; OA = Oropharyngeal aspiration; PBS= Phosphate Buffered Saline; SAP = Severe Acute Pancreatitis; SCID = Severe combined immunodeficiency
