# Supplementary material for: Efficacy of Mesenchymal Stromal Cell Therapy for Acute Lung Injury in Preclinical Animal Models: A Systematic Review
Source: PLoS One. 2016 Jan 28;11(1):e0147170. doi: 10.1371/journal.pone.0147170 (PMC4731557; doi:10.1371/journal.pone.0147170)
Supplement: S2 Table — (DOCX) [file pone.0147170.s003.docx]

**S2 Table**: Cochrane Risk of Bias Assessment

| **Author (Year)** | **Random Sequence Generation** | **Allocation Concealment** | **Blinding of Personnel** | **Blinding of Outcome Assessment:**  **Mortality** | **Incomplete Outcome Data** | **Selective Reporting** | **Other Bias** |
| --- | --- | --- | --- | --- | --- | --- | --- |
| Gupta (2007)A^7^ | Unclear | Unclear | Unclear | Low# | Unclear | Low | Unclear |
| Gupta (2007)B^7^ | Unclear | Unclear | Unclear | Low# | Unclear | Low | Unclear |
| Gonzalez-Rey (2009)A^46^ | Unclear | Unclear | Unclear | Low# | Unclear | Low | High |
| Gonzalez-Rey (2009)B^46^ | Unclear | Unclear | Unclear | Low# | Unclear | Low | High |
| Leblond (2009)^44^ | Unclear | Unclear | Unclear | Low# | Unclear | High | Unclear |
| Nemeth (2009)^47^ | Unclear | Unclear | Unclear | Low* | Unclear | Low | Unclear |
| Bi (2010)^59^ | Unclear | Unclear | Unclear | Unclear | Unclear | Low | Unclear |
| Lee (2010)^43^ | Unclear | Unclear | Unclear | Unclear | Unclear | High | Unclear |
| Mei (2010)A^41^ | Unclear | Unclear | Unclear | Low* | Unclear | Low | High |
| Mei (2010)B^41^ | Unclear | Unclear | Unclear | Low* | Unclear | Low | High |
| Kim (2011)^10^ | Unclear | Unclear | Unclear | Low# | High | Low | Unclear |
| Liang (2011)^34^ | Unclear | Unclear | Unclear | Low* | Low | Low | Unclear |
| Sun (2011)#1^37^ | Unclear | Unclear | Unclear | Unclear | Unclear | Low | Unclear |
| Chang (2012)^17^ | Unclear | Unclear | Unclear | Unclear | Low | Low | Low |
| Gupta (2012)^29^ | Unclear | Unclear | Unclear | Low# | Unclear | Low | Unclear |
| Krasnodembskaya (2012)B^31^ | Unclear | Unclear | Unclear | Low# | Unclear | Low | Unclear |
| Li (2012)B^15^ | Unclear | Unclear | Unclear | Unclear | Low | Low | Unclear |
| Wu (2012)#1C^32^ | Unclear | Unclear | Unclear | Unclear | Low | Low | Unclear |
| Yang (2013)#1A^18^ | Unclear | Unclear | Unclear | Low# | Low | Low | Unclear |
| Yang (2013)#1B^18^ | Unclear | Unclear | Unclear | Low# | Low | Low | Unclear |
| Zhao (2013)^16^ | Unclear | Unclear | Unclear | Low# | Low | Low | Unclear |

**Legend:** Numbers following study author and year (ex. Wu 2012 #1) indicate that the author published more than one paper in the same year that is included in this systematic review. Letters following author and year (ex. Gupta 2013A) indicate that more than one experiment was conducted in the same publication that reported mortality.

**Blinding of Outcome Assessment for Mortality**: Low risk = Outcome assessors were blinded to the study groups when assessing mortality (indicated by *), or animals were allowed to die (indicated by #). Unclear = Insufficient information to determine if outcome assessors were blinded, or if animals were allowed to die. High Risk = Outcome assessors not blinded to the study groups and death was defined according to pre-specified physiological criteria.

**Incomplete Outcome Data**: Low risk = N values were consistent between methods and results for the mortality outcome. Unclear = The N value was either not presented in the methods or in the results, and therefore there is insufficient information to permit judgement. High risk = N values were not consistent between methods and results for the mortality outcome.

**Selective Reporting**: Low risk = The methods section indicated mortality as a pre-specified outcome measure. High risk = The mortality outcome was presented in the results but not pre-specified in the methods section.
