# Supplementary material for: Efficacy of Mesenchymal Stromal Cell Therapy for Acute Lung Injury in Preclinical Animal Models: A Systematic Review
Source: PLoS One. 2016 Jan 28;11(1):e0147170. doi: 10.1371/journal.pone.0147170 (PMC4731557; doi:10.1371/journal.pone.0147170)
Supplement: S3 Table — (DOCX) [file pone.0147170.s004.docx]

**S3 Table**: Cochrane “Other” Risk of Bias Assessment

| **Author (Year)** | **Source of Funding** | **Sample Size Calculation** | **Conflict of Interest** |
| --- | --- | --- | --- |
| Gupta (2007)A^7^ | Low | Unclear | Low |
| Gupta (2007)B^7^ | Low | Unclear | Low |
| Gonzalez-Rey (2009)A^46^ | High | Unclear | Unclear |
| Gonzalez-Rey (2009)B^46^ | High | Unclear | Unclear |
| Leblond (2009)^44^ | Low | Unclear | Low |
| Nemeth (2009)^47^ | Low | Unclear | Unclear |
| Bi (2010)^59^ | Low | Unclear | Unclear |
| Lee (2010)^43^ | Unclear | Unclear | Low |
| Mei (2010)A^41^ | High | Unclear | High |
| Mei (2010)B^41^ | High | Unclear | High |
| Kim (2011)^10^ | Low | Unclear | Low |
| Liang (2011)^34^ | Low | Unclear | Unclear |
| Sun (2011)#1^37^ | Low | Unclear | Unclear |
| Chang (2012)^17^ | Low | Low | Low |
| Gupta (2012)^29^ | Low | Unclear | Low |
| Krasnodembskaya (2012)B^31^ | Low | Unclear | Low |
| Li (2012)B^15^ | Low | Unclear | Low |
| Wu (2012)#1C^32^ | Low | Unclear | Unclear |
| Yang (2013)#1A^18^ | Unclear | Unclear | Low |
| Yang (2013)#1B^18^ | Unclear | Unclear | Low |
| Zhao (2013)^16^ | Low | Unclear | Low |

**Legend:** Numbers following author and year (ex. Wu 2012 #1) indicate that the first author published more than one paper in the same year that are included in this systematic review.

Other risk of bias was assessed according to source of funding, conflict of interest and pre-specified sample size calculations:

**Source of Funding**: Low risk = Non-industry source of funding (or no funding), Unclear = Funding source was not reported, High risk = Study was funded by industry

**Conflict of Interest**: Low risk = Authors reported on no conflict of interest, Unclear = conflict of interest was not reported, High risk = Authors reported on potential conflict of interests

**Sample Size Calculation**: Low risk = Sample size calculations were correctly performed and followed, Unclear = sample size calculations were not performed, High risk = Sample size calculations were incorrectly performed/followed

Overall risk for the domain was determined as follows: if any one of the components were determined to be of high risk, the overall domain was assessed as high risk. The domain could only be assessed as low risk is all three components were determined to be of low risk.
