# Supplementary material for: Efficacy of Mesenchymal Stromal Cell Therapy for Acute Lung Injury in Preclinical Animal Models: A Systematic Review
Source: PLoS One. 2016 Jan 28;11(1):e0147170. doi: 10.1371/journal.pone.0147170 (PMC4731557; doi:10.1371/journal.pone.0147170)
Supplement: S4 Table — (DOCX) [file pone.0147170.s005.docx]

**S4 Table**: Criteria for identification of cell population as human mesenchymal stromal cells

| **Author (Year)** | **Does study report the MSC criteria are met?** | **Plastic-adherent in standard culture conditions?** | **Positive marker** | **Value given (%)*** | **Negative marker** | **Value given (%)*** | **Were MSC supplied?** | **Differentiation capability** |
| --- | --- | --- | --- | --- | --- | --- | --- | --- |
| Gonzalez-Rey (2009)^46^ | N | U | CD73, CD90, CD105 | NR | CD14, CD34, CD45 | NR | N | O, A |
| Moodley (2009)^45^ | N | Y | CD73 | NR | CD34, CD45 | NR | N | O |
| Bi (2010)^59^ | N | Y | CD105, | NR | CD45 | NR | N | NR |
| Krasnodem-bskaya (2010)^11^ | Y | U | NR | N/A | CD45, CD19 | NR | Y, Texas A&M Health Science Center College of Medicine, Bryan, Texas | NR |
| Yagi (2010) #1^40^ | N | U | NR | N/A | NR | N/A | Y, Tulane Center for Gene Therapy, New Orleans, Louisiana | NR |
| Yagi (2010) #2^42^ | N | U | NR | N/A | NR | N/A | Y, Tulane Center for Gene Therapy, New Orleans, Louisiana | O, A |
| Danchuk (2011)^13^ | N | U | NR | N/A | NR | N/A | Y, Center for the Preparation and Distribution of Adult Stem Cells, Institute for Regenerative Medicine, Texas A&M Health Sciences Center, Bryan, Texas | NR |
| Kim (2011)^10^ | N | Y | CD105, CD73 | 99.6, 96.3, | CD34, CD45, CD14 | 0.1,  0.2,  0.1 | N | O, A, C |
| Sun (2011) #1^37^ | N | Y | CD73 | NR | CD45, CD34, | NR | N | O, A |
| Chien (2012)^27^ | N | U | NRMR | NR | NRMR | N/A | N | NR |
| Krasnodem-bskaya (2012)^31^ | Y | U | NR | N/A | CD45, CD19 | NR | Y, Texas A&M Health Science Center College of Medicine, Bryan, Texas | NR |
| Li (2012)^15^ | N | Y | CD73, CD90, CD105 | 99.74, 99.1, 84.1 | CD34, CD45 | 0.4,  0.1 | N | O, A |
| Wu (2012)#2^53^ | N | U | NR | N/A | NR | N/A | N | NR |
| Gao (2013)^52^ | N | U | CD90 | 90.6 | CD34 | 1.8 | N | NR |
| Lim (2013)^14^ | N | U | NR | N/A | NR | N/A | Y, Tulane Center for Gene Therapy, New Orleans, Louisiana | NR |
| Shin (2013)^12^ | N | Y | CD90, CD105 | 98.5, 39.7 | CD34 | <1 | N | NR |
| Yang (2013) #1^18^ | N | U | CD73, CD90, CD105 | 99.28, 100, 99.37 | CD19, CD34, CD45, CD11b, HLA-DR | 0.15, 0.09, 0.02, 0.77, 0.96 | Y, State Stem Cell Industry Base, Tianjin, China | NR |
| Zhang (2013)^57^ | N | Y | NRMR | NR | CD11b, CD45 | NR | N | O, A |

Abbreviations: A=adipocyte, C=chondroblast, N=no, N/A=not applicable, NR= not reported, NRMR=no relevant marker reported, O=Osteoblast, U=unclear, Y=yes

*Minimum MSC criteria (Dominici, 2006) specify ≥95% of the cells must express CD105, CD73 and CD90 while ≤2% of cells can express CD45, CD34, CD14 or CD11b, CD79α or CD19, and HLA-DR
